# MLP Enhanced CO_2_ Emission Prediction Model with LWSSA Nature Inspired Optimization

**DOI:** 10.1038/s41598-025-85709-5

**Published:** 2025-01-13

**Authors:** Agoub Abdulhafith Younes Mussa, Wagdi M. S. Khalifa

**Affiliations:** Cyprus Health and Social Sciences University, Mersin 10, Turkey

**Keywords:** Machine Learning, Carbon Emission, Multi-layer Perceptron, Optimization, Global Trade, Computer science, Information technology

## Abstract

Environmental degradation due to the rapid increase in CO₂ emissions is a pressing global challenge, necessitating innovative solutions for accurate prediction and policy development. Machine learning (ML) techniques offer a robust approach to modeling complex relationships between various factors influencing emissions. Furthermore, ML models can learn and interpret the significance of each factor’s contribution to the rise of CO_2_. This study proposes a novel hybrid framework combining a Multi-Layer Perceptron (MLP) with an enhanced Locally Weighted Salp Swarm Algorithm (LWSSA) to address the limitations of traditional optimization techniques, such as premature convergence and stagnation in locally optimal solutions. The LWSSA improves the standard Salp Swarm Algorithm (SSA) by incorporating a Locally Weighted Mechanism (LWM) and a Mutation Mechanism (MM) for greater exploration and exploitation. The LWSSA-MLP framework achieved a prediction accuracy of 97% and outperformed traditional optimizer-based MLP models across several evaluation metrics. A permutation feature significance analysis identified global trade, coal energy, export levels, urbanization, and natural resources as the most influential factors in CO₂ emissions, offering valuable insights for targeted interventions. The study provides a reliable and scalable framework for CO₂ emission prediction, contributing to actionable strategies for sustainable development and environmental resilience.

## Introduction

Human innovation and technological advancement have long been catalysts for societal progress. However, this trajectory of development has come at a significant cost: the stability of the planet’s climate^1^. Climate change, largely driven by greenhouse gas emissions, is a global crisis manifesting in extreme weather events, biodiversity loss, and adverse health impacts. Among these gases, CO₂ accounts for over 60% of total emissions, primarily due to the burning of fossil fuels^2^^3^. Addressing this crisis necessitates the development of accurate, data-driven frameworks to forecast emissions and inform policy decisions. Historically, the severity of climate change went unrecognized despite early warnings, such as the 1960 study by the central intelligence agency highlighting the potential for significant climatological changes^4^^5^. By the late 1980s, the establishment of the Intergovernmental Panel on Climate Change (IPCC) catalyzed efforts to coordinate global scientific collaboration, emphasizing the urgency of mitigating emissions. The adverse impacts of CO₂ emissions include rising global temperatures, prolonged droughts, and significant health risks, underscoring the importance of precise forecasting to guide mitigation strategies^6^.

ML has emerged as a powerful tool for CO₂ emission prediction due to its ability to model complex, non-linear relationships in data. ML has been applied in several fields, demonstrating superior prediction performance^7,8^. Several studies have demonstrated the efficacy of ML in the emission prediction domain. For instance, Tripathi et al. employed Artificial Neural Networks (ANN) to predict CO₂ conversion rates, achieving high accuracy and uncovering hidden catalytic correlations^9^. Nguyen et al. explored the potential of supervised and unsupervised learning methods, such as Deep Belief Networks (DBN) and Convolutional Neural Networks (CNN), for CO₂ monitoring, demonstrating their superiority over traditional statistical models^10^. Li and Zhang evaluated six models for real-time daily CO₂ emission predictions in China, covering data from January 2020 to September 2022. The study compared three traditional statistical models, Grey Prediction, Auto Regressive Integrated Moving Average (ARIMA), and Seasonal Auto-Regressive Integrated Moving Average with Exogenous factors (SARIMAX), with three machine learning models ANN, Long Short-Term Memory (LSTM) and Random Forest (RF). ML models outperformed statistical models, with LSTM achieving the highest accuracy and robustness, as assessed by metrics like Mean Square Error (MSE), Root Mean Square Error (RMSE), and Coefficient Determinant (R^2^)^11^. Similarly, Singh et al. analyzed four forecasting models, SARIMAX, RF, Holt-Winters, and Support Vector Regression (SVR), to predict CO₂ emissions from paddy crops in India, using data from 1961 to 2018. They found Holt-Winters and SVR to be the most accurate, providing reliable forecasts for 2025^12^.

Gina et al. developed a Multi-Layer Artificial Neural Network (MANN) to forecast CO₂ emissions across 17 countries. Their proposed model demonstrated an average accuracy of 96% compared to traditional linear statistical methods^13^. Furthermore, Hassan et al. developed an Improved Opposition-Based Particle Swarm Optimization algorithm integrated with an ANN. The introduction of the improved optimization algorithm enhanced the ANN’s training process. The results demonstrated that the proposed model outperformed traditional methods, yielding superior outcomes^14^.

Over the years, the MLP has emerged as one of the most well-established ML models, finding applications in various domains and in CO_2_ prediction. Nanda et al. optimized the MLP using the Modified Coyote Optimization Algorithm (MCOA). Their experimental results indicated that the MLP-MCOA outperformed LSTM, k-Nearest Neighbors (KNN), and CNN in terms of accuracy^15^. Similarly, Adegboye et al. enhanced an MLP model by employing the Worst Moth Disrupted Moth Fly Optimization (WMFO) algorithm to fine-tune its weights and biases. The resulting WMFO-MLP model achieved a remarkable accuracy of 97.8%, surpassing other optimization-based models^16^. Rostami et al. utilized an MLP model for the precise estimation of CO₂ adsorption on activated carbon, demonstrating the model’s efficacy in CO₂ adsorption prediction application^17^. Likewise, Bastani et al. developed an MLP model for predicting the CO₂ loading capacity of chemical absorbents. Their comparative analysis with other established models highlighted the superiority of the MLP approach^18^. Moayedi et al. combined the MLP with the Teaching–Learning-Based Optimization (TLBO) and Vortex Search Algorithm (VSA), yielding significant improvements in CO₂ emission prediction accuracy^19^.

Traditional ML models often face inherent limitations, such as their reliance on initial weights and biases and parameter tuning problems^19,20^. These issues become particularly pronounced in complex, non-linear problems like CO₂ emission prediction, where the ability to explore diverse solutions is critical. Such limitations can lead to reduced accuracy and a diminished capacity to generalize, ultimately affecting the reliability of predictions. To address these challenges, researchers have increasingly turned to hybrid models that integrate ML with nature-inspired optimization algorithms. These hybrid approaches aim to efficiently fine-tune the parameters of ML models, thereby improving their accuracy and robustness. Sahraei and Çodur utilized ANN enhanced with Particle Swarm Optimization (ANN-PSO) to optimize energy demand predictions, effectively mitigating the limitations of traditional ML models and showcasing the potential of these integrated frameworks to deliver more precise and reliable outcomes^21^. Emami Javanmard and Ghaderi predicted greenhouse gas emissions in Iran (1990–2018) using nine ML algorithms and enhanced forecasting accuracy by integrating PSO and Grey Wolf Optimizer (GWO) into the model, achieving improvements of 31.7% and 12.8%, respectively^22^. Khajavi and Rastgoo applied a hybrid approach combining RF, SVR, and Response Surface Methodology (RSM) to predict CO₂ emissions in 30 major Chinese cities. By tuning hyperparameters with various optimizers, the SVR enhanced Harris Hawk Optimizer (HHO) had the highest training accuracy, while RF optimized Slime Mould Algorithm (SMA) achieved the best testing accuracy^23^. Moayedi et al. improved the prediction accuracy of MLP by optimizing them with Shuffled Complex Evolution (SCE) and Biogeography-Based Optimization (BBO), showing significant accuracy in CO₂ emission forecasting^24^. Adegboye et al. proposed a Support Vector Regression (SVR) model fine-tuned using the Sine Cosine Perturbation with Chaotic Perturbation and Mirror Imaging Strategy-based Salp Swarm Algorithm (SCMSSA). Their experimental results demonstrated improved accuracy in CO₂ prediction^25^. Zhao et al. introduced a hybrid model combining the Whale Optimization Algorithm (WOA) with the Least Squares Support Vector Machine (LSSVM), referred to as the WOA-LSSVM model. The WOA was employed to optimize two key parameters of the LSSVM, resulting in enhanced accuracy during CO₂ prediction^26^. Wen and Cao developed an enhanced Butterfly Optimization Algorithm (BOA) to optimize the parameters of the LSSVM. Their hybrid model was applied to predict residential CO₂ emissions, with results indicating significant improvements in prediction accuracy^27^

The aforementioned studies highlight the growing trend of integrating ML models with nature-inspired optimization algorithms to enhance predictive accuracy and robustness, particularly in CO₂ emission prediction. While these hybrid models have shown significant improvements in overcoming traditional ML limitations, such as reliance on initial weights and biases or hyper parameter tuning, several gaps remain. For instance, the models often rely on specific datasets, limiting their generalizability. Additionally, while methods like PSO, GWO, HHO, and SMA improve performance, many of these original algorithms struggle to balance exploration and exploitation effectively, leading to premature convergence in complex, multi-modal problems, yielding less accuracy^28^. Furthermore, these studies focus solely on improving prediction metrics without providing comprehensive feature importance analyses, leaving a gap in understanding the factors driving emissions.

Therefore, this study addresses these gaps by proposing a novel hybrid framework combining MLP with the Locally Weighted Salp Swarm Algorithm (LWSSA)^29^. The MLP, which is a type of ML known for its capability to handle complex, non-linear relationships, serves as the predictive algorithm of the framework. MLP learns by adjusting the weights and biases of its neurons through a self-assignment process. It performs well on large input data, and provides fast predictions once trained. However, its performance is often hindered by reliance on weights and biases, which is addressed by introducing LWSSA as an optimization strategy. The LWSSA enhances the standard SSA through two key mechanisms: LWM to steer the search process toward high-quality solution regions and a Mutation Operator MM to increase randomness and diversity, ensuring a better balance between exploration and exploitation. The contributions of this study are as follows:

1. Leveraging the enhanced LWSSA to overcome the limitations of traditional SSA, such as premature convergence and susceptibility to local optima, improves its suitability for optimizing CO₂ emission prediction tasks.
2. Develop a hybrid framework by integrating the adapted LWSSA with the MLP to address MLP’s limitations, including sensitivity to weights and biases. This integration enhances prediction accuracy and generalization capacity in complex, non-linear problems such as CO₂ emission forecasting.
3. Conduct extensive experiments to evaluate the performance of the proposed LWSSA against established algorithms on the Congress on Evolutionary Computation (CEC2015) benchmark problems. Assessing the LWSSA, to establish the enhanced optimization capability of LWSSSA. Furthermore, evaluation of the LWSSA-MLP framework in predicting CO_2_ emission in comparison to other optimizer-enhanced MLP models using several metrics.
4. Incorporating a permutation-based feature importance assessment within the LWSSA-MLP framework to identify and understand key factors influencing CO₂ emissions. This analysis offers actionable insights for policymakers, highlighting the critical drivers of emissions and their relative contributions.

By addressing these limitations and providing a comprehensive framework, this study not only improves CO₂ emission prediction accuracy but also enhances the interpretability of contributing factors, filling the identified gaps in existing research.

The organization of this paper is as follows: Sect. 2 provides essential background information on the original SSA. Section 3 introduces the LWSSA optimizer. Section 4 presents the integration of LWSSA with MLP. Section 5 discusses the experiments conducted on CO_2_ prediction and their results. Finally, Sect. 6 offers concluding remarks and summarizes the key findings of this study.

## Background

### Salp Swarm algorithm (SSA)

The salp, a member of the Salpidae family, bears a resemblance to jellyfish and feeds through internal filters^30,31^. When salps aggregate to form chains, their foraging efficiency and locomotion are significantly enhanced. This swarming behavior is a common phenomenon among various marine species. Inspired by this natural behavior, the SSA has been developed as a meta-heuristic optimization technique. The SSA models the coordinated movement of salps within a swarm, with a designated leader guiding the followers in their exploration and exploitation of the search space. The swarm of salps is mathematically represented by a two-dimensional matrix, $X$, as expressed in Eq. (1). The fitness of each salp is evaluated to identify the optimal individual, which is then designated as the leader of the swarm. Subsequently, the salp population is divided into two, namely leaders and followers. The positions of leader salps are updated based on the formulation provided in Eq. (2).

1 $$x_{i} = \left[ {\begin{array}{*{20}c} {X_{1}^{1} } & {X_{1}^{1} } & \cdots & {X_{1,d}^{1} } \\ {X_{1}^{2} } & {X_{2}^{2} } & \cdots & {X_{d}^{2} } \\ \vdots & \ddots & \ddots & \vdots \\ {X_{1}^{n} } & {X_{d}^{n} } & \cdots & {X_{d}^{n} } \\ \end{array} } \right]$$

2 $${X}_{i}^{1}=\left\{\begin{array}{cc}yi+{r}_{1}*\left(\left(u{b}_{i}-l{b}_{i}\right)r2+l{b}_{i}\right);& r3<0.5\\ yi- {r}_{1}*\left(\left(u{b}_{i}-l{b}_{i}\right)r2+l{b}_{i});\right.& r3\ge 0.5\end{array}\right.$$

${y}_{i}$ denotes the location of a food source in the *i*-th dimension, while ${x}_{i}{ }^{1}$ represents the position of a leader salp in the same dimension. The coefficient ${r}_{1}$, as defined in Eq. (3). Equation (2) incorporates the lower $l{b}_{i}$ and upper $u{b}_{i}$ bounds of the dimension to guide the search process. Additionally, the coefficients ${r}_{2}$ and ${r}_{3}$ are randomly generated values within the range of 0 to 1, ensuring stochasticity in the optimization process.

3 $${r}_{1}={2\text{e}}^{-{\left(\frac{4I}{L}\right)}^{2}}$$

The variable $I$ signifies the current iteration, while *L* denotes the maximum number of iterations. The coefficient ${r}_{1}$ is pivotal in the SSA as it regulates the balance between exploration and exploitation throughout the optimization process. The updated positions of the follower salps are determined using the formulation provided in Eq. (4).

4 $${x}_{i}^{j}=\frac{1}{2}\lambda {t}^{2}+{\delta }_{0}t$$

The position of the *n*-th salp in the *i*-th dimension is represented by ${x}_{i}{ }^{1}$. Here, $t$ denotes time, while ${\delta }_{0}$ and ${\delta }_{\text{final}}$ represent the initial and final speeds, respectively. These values are calculated using the expression provided in Eq. (5).

5 $$\lambda = \frac{{\delta_{final } }}{{\delta_{0} }}\,\,,\,\,\delta_{0} = \frac{{x - x_{0} }}{t}$$

With ${\delta }_{0}$=0, Eq. (4) can be rewritten as Eq. (6):

6 $${x}_{i}^{j}=\frac{1}{2}\left({x}_{i}^{j}+{x}_{i}^{j-1}\right)$$

Here, ${x}_{i}^{j}$ denotes the position of the *j*-th follower salp in the *i*-th dimension. The parameter ${r}_{1}$ is adaptively decreased as the iterations progress, enabling the SSA to balance exploration and exploitation. This mechanism allows the algorithm to thoroughly explore the search space during the initial stages and subsequently concentrate on refining solutions in promising regions. If a salp moves outside the boundaries of the search space, it is repositioned using the corrective mechanism defined in Eq. (7).

7 $${x}_{i}^{j}=\left\{\begin{array}{c}l{b}_{i}\hspace{0.25em}\hspace{0.25em}\hspace{0.25em}\hspace{0.25em} \, {\text{i}}{\text{f}} \, {x}_{i}^{j}\le l{b}_{i}\\ u{b}_{i}\hspace{0.25em}\hspace{0.25em}\hspace{0.25em}\hspace{0.25em} \, {\text{i}}{\text{f}} \, {x}_{i}^{j}\ge u{b}_{i}\\ {x}_{i}^{j}\hspace{0.25em}\hspace{0.25em}\hspace{0.25em}\hspace{0.25em} \, {\text{o}}{\text{t}}{\text{h}}{\text{e}}{\text{r}}{\text{w}}{\text{i}}{\text{s}}{\text{e}}\text{.}\end{array}\right.$$

## Locally weighted salp swarm algorithm (LWSSA)

### Locally weighted mechanism (LWM)

The locally weighted technique is a heuristic approach designed to address complex optimization problems. This method involves iteratively integrating a neighboring solution from the search space into the current solution, as described in^29^. A key challenge in local search algorithms is the selection of appropriate neighbors from a potentially infinite set of options, which is critical for achieving optimal results. The Local Search Algorithm (LWA) employs this local search strategy to enhance the current solution at each iteration of the optimization process. SSA optimizes a population $x$, consisting of $j$ salps and one solution ${x}_{i}^{j}=\left({x}_{i}^{1},{x}_{i}^{2},\dots {x}_{dim}^{j}\right)$ to generate an updated solution ${x}_{i}^{new}$. Subsequently, the LWA further refines the salp’s position, producing ${y}_{i}^{new}$, as determined by Eqs. (8) and (9).

8 $${\text{weight }}_{j}=\frac{1}{\left(1+\text{exp}\left({x}_{i}^{new}-{x}_{i}^{j}\right)\right)}$$

9 $${y}_{i}^{new}={x}_{i}^{new}+Z\times \left({\text{ weight }}_{j}\times \left({x}_{i}^{{r}_{1}}-{x}_{i}^{{r}_{2}}\right)\right)$$

Two particles, ${x}_{i}^{{r}_{1}}$ and ${x}_{i}^{{r}_{2}}$, are randomly selected from the population $j$, excluding the current particle ${x}_{i}^{j}$. Furthermore, *Z* is a random integer generated using the formulation provided in Eq. (10). This generation process employs the magenta technique, which is based on the Lévy distribution, as described in^32^.

10 $$z=0.01\times \frac{b}{|q{|}^{\frac{1}{\alpha }}}$$

Here, $\beta$ is determined using Eq. (11), while the parameters $b$ and $q$ are sampled from normal distributions. Specifically, $b\sim N\left(0,{\beta }^{2}\right)$ and $q\sim N\left(0,{\beta }^{2}\right)$, ensuring randomness in the optimization process guided by the specified distribution parameters.

11 $$\beta =\sqrt[\alpha ]{\frac{\Gamma (1+\alpha )\text{sin}\left(\frac{\pi \alpha }{2}\right)}{\Gamma \lceil\frac{\alpha +1}{2}\rceil\alpha {2}^{\frac{\alpha -1}{2}}}}$$

where the index of stability *α* (Levy index ) is taken from [0, 2].

### Mutation mechanism (MM)

The SSA offers several advantages, including adaptability, simplicity in implementation, and a reduced number of parameters compared to other optimization algorithms. However, its effectiveness in achieving global optima diminishes over successive iterations due to stagnation, which arises from constant updates to the leader’s position. Furthermore, the mathematical model of SSA does not explicitly address the balance between exploration and exploitation, raising concerns about its performance in high-dimensional optimization problems. To mitigate the issue of local stagnation, Mohammed et al. proposed a modification to the formula used for updating the positions of followers in a salp chain. This enhancement incorporates a mutation factor, as defined in Eq. (12), to introduce additional diversity and improve the algorithm’s global search capabilities^29^.

12 $${y}_{i}^{new}={x}_{i}^{j}+\text{ rand }\times mu\times \left({x}_{i}^{{r}_{1}}-{x}_{i}^{{r}_{2}}\right)$$

The constant mutation factor, $mu$, is set to 0.5, while the variable $rand$ represents a randomly generated value within the range of 0 to 1. The variables ${x}_{i}^{{r}_{1}}$ and ${x}_{i}^{{r}_{2}}$ denote two randomly selected positions within the population, excluding ${x}_{i}^{j}$. These variables acquire information about the positions of other individuals relative to ${x}_{i}^{j}$. When there is a significant disparity between ${x}_{i}^{{r}_{1}}$ and ${x}_{i}^{{r}_{2}}$, the updated individual is more likely to adjust its position toward the midpoint of this range, thereby enhancing exploration of the search space. Conversely, when the proximity between ${x}_{i}^{{r}_{1}}$ and ${x}_{i}^{{r}_{2}}$ is minimal, the updated individual tends to focus on searching within its immediate vicinity, fostering exploitation. The incorporation of the $mu$ principle facilitates a collective optimization strategy, reducing positional disparities across individuals without relying solely on the efforts of a single member. Additionally, a random value between 0 and 1 is integrated to modify the position of the food source, leveraging the inherent stochasticity of the SSA, as described in Eq. (2). This approach mitigates the risk of stagnation in local optima and ensures the continuity of the convergence process.

The subsequent section provides a detailed explanation of the optimization scenario implemented in the proposed algorithm.

### LWSSA work flow

**Fig. 1:**
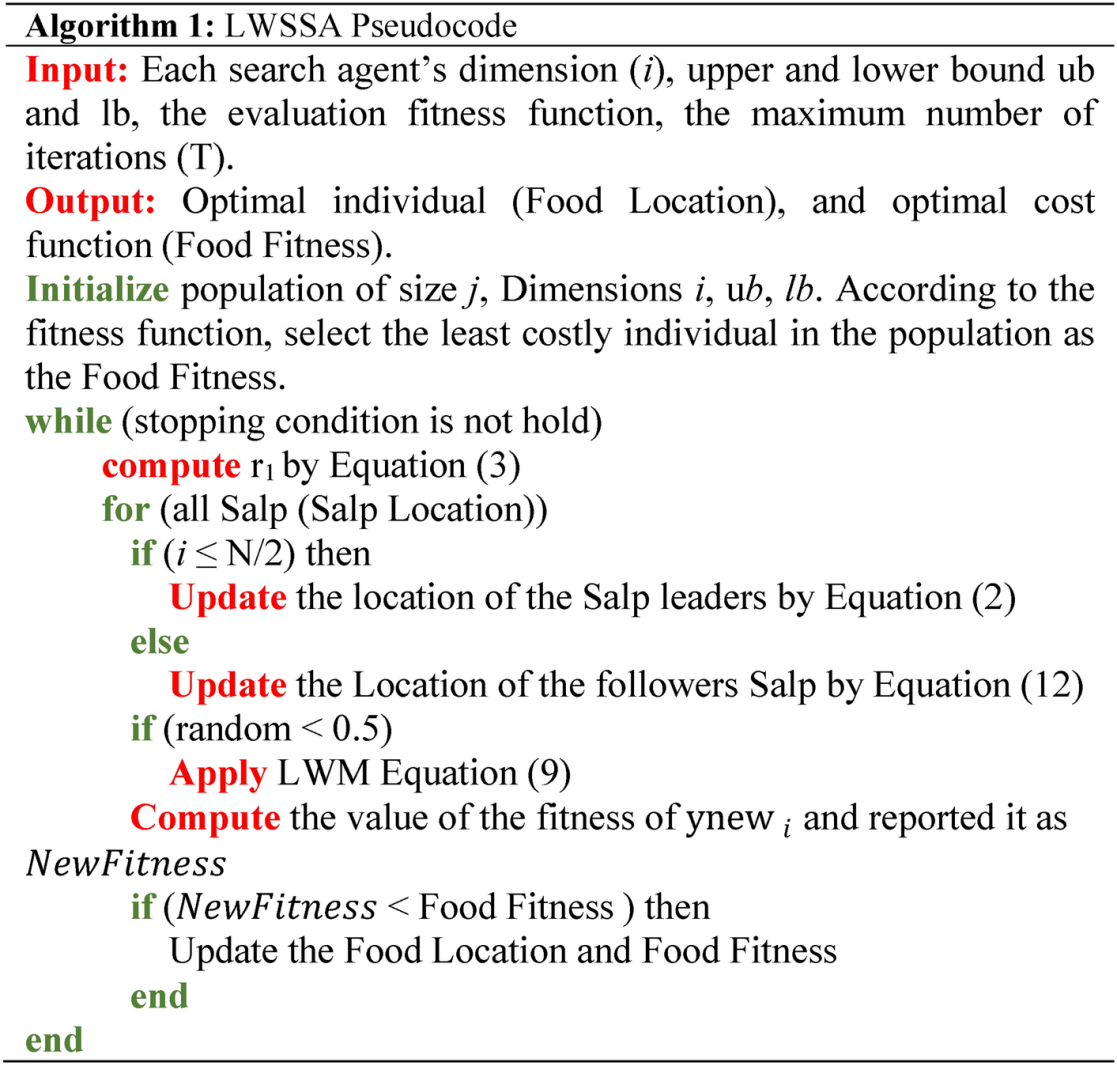
LWSSA Algorithm Pseudocode.

**Fig. 2:**
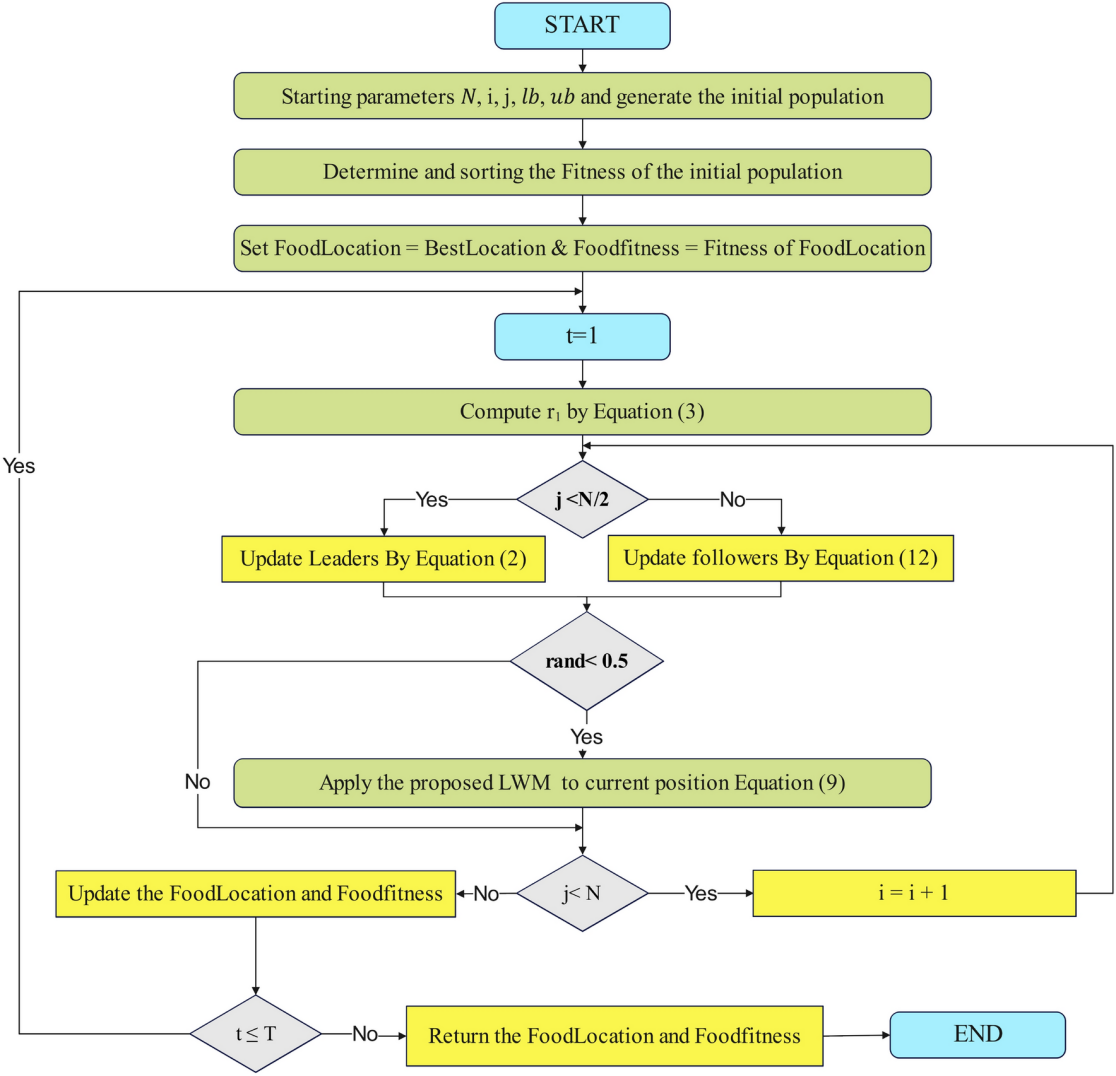
LWSSA Algorithm Flow Chart.

The LWSSA algorithm, as depicted in Fig. 1, integrates the SSA with the LWM and MM to efficiently identify optimal solutions while addressing the risk of entrapment in local minima. These mechanisms complement each other to improve the algorithm’s performance through distinct but synergistic roles. The algorithm begins by dividing the population into two groups. The first group, comprising the initial half of the population (leaders), updates its positions using Eq. (2), which facilitates an exploratory search for potential solutions. The second group, or followers, employs the MM, defined in Eq. (12), to update positions. MM enhances the diversity of the search by introducing controlled randomness. This process allows followers to escape from local optima and explore uncharted regions of the search space. The random nature of MM ensures that the algorithm does not prematurely converge to suboptimal solutions, thereby improving its global search capabilities. Following this, the LWM is applied to refine solutions and determine optimal positions for individuals. LWM focuses on intensifying the search around promising regions identified by the SSA and MM. By adjusting positions with a probability of 0.5 for the entire population, LWM balances exploration and exploitation. This probabilistic refinement ensures that the algorithm not only diversifies its search across the problem space but also concentrates resources on refining high-potential areas. This balance is critical for achieving a near-optimal solution efficiently. Both mechanisms, MM and LWM, improve the algorithm in complementary ways. MM ensures sufficient exploration by mitigating the risk of stagnation and enabling the algorithm to traverse complex, multi-modal landscapes effectively. Meanwhile, LWM enhances exploitation by fine-tuning solutions in promising regions, accelerating convergence towards the global optimum. Together, these mechanisms create a dynamic balance that allows LWSSA to outperform traditional SSA and other optimization algorithms. The procedural steps of this improved method are outlined in Fig. 2, offering a comprehensive overview of the workflow and the mechanisms’ roles in achieving enhanced optimization performance. This combination of exploration and exploitation strategies ensures robust performance across diverse optimization scenarios.

### Complexity of LWSSA

The computational complexity of the LWSSA arises from three main components SSA, the MM, and the LWM. In SSA, the position updates for leader and follower salps require operations proportional to the population size (${N}_{\text{pop}}$) and the problem dimensionality ($D$), resulting in a complexity of $O\left({N}_{\text{pop}}\times D\right)$ per iteration. The mutation operator, applied to followers, adds a similar cost of $O\left({N}_{\text{pop }}\times D\right)$. Additionally, the LWM, which refines solutions probabilistically, contributes $O\left({N}_{\text{pop }}\times D\right)$, assuming an update probability of 0.5. Combining these components, the overall complexity per iteration is $O\left(3\times {N}_{\text{pop }}\times D\right)$, which simplifies to $O\left({N}_{\text{pop }}\times D\right)$. For a total of $T$ iterations, the algorithm’s total complexity is $O(T\times$ ${N}_{\text{pop }}\times D$ ). This linear complexity with respect to population size and dimensionality ensures computational efficiency for medium-scale problems. While the cost grows proportionally with dimensionality.

## LWSSA-MLP prediction model

### Multi-Layer perceptron (MLP)

**Fig. 3:**
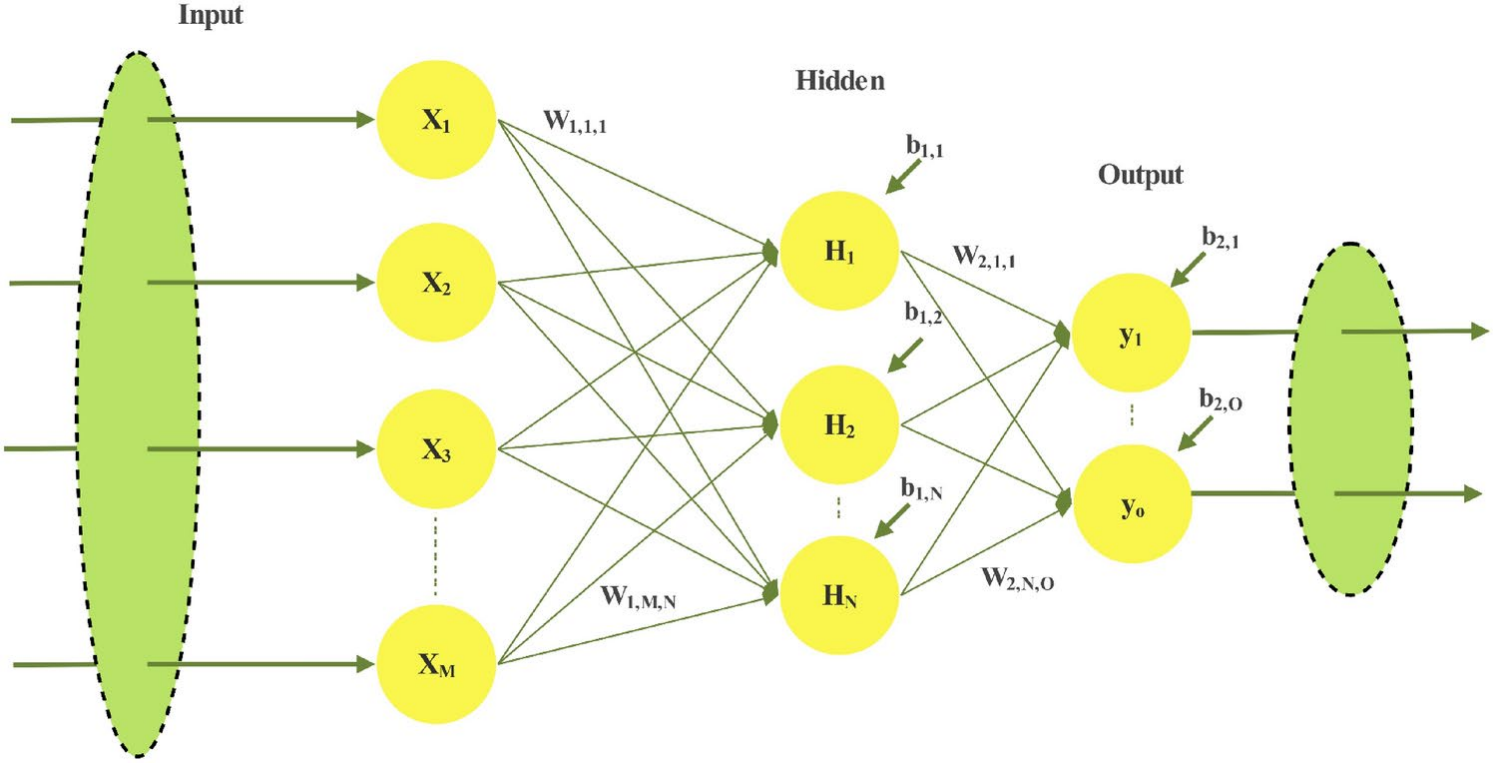
Simple Multi-Layer Perceptron.

Feedforward Neural Networks (FNNs) consist of interconnected neurons arranged across multiple layers, with unidirectional synapses facilitating the flow of information between neurons. Among the various types of FNNs, the MLP is a widely utilized and distinct variant. In an MLP, neurons are distributed across multiple densely connected layers, which are classified into three primary categories: input layers, hidden layers, and output layers^33^. The input layer of the network receives data from the dataset and serves as the entry point, channeling this information into the network for processing. The output layer, positioned at the end, generates the network’s final results. Situated between these two are the hidden layers, which perform intermediate computations to extract and transform features. Figure 3 depicts a simple MLP model with a single hidden layer. This MLP structure consists of three layers: an input layer containing *M* neurons, a hidden layer with *N* neurons, and an output layer comprising *O* neurons.

In addition to the distinct layers, three fundamental components define the specifications of a neural network: the connections between neurons, represented by biases and weights, and the activation functions, which are critical in determining the outputs of the MLP neurons within the hidden and output layers^33^. The output of the MLP is computed through a series of mathematical operations, beginning with the calculation of the weighted sum of the inputs, as described by the summation equation in Eq. (13).

13 $${u}_{j}=\sum_{i=1}^{P} {\omega }_{1,i,j}\cdot {x}_{i}-{b}_{1,j},j=\text{1,2},\cdots N$$

${x}_{i}$ represents the *i*-th input, ${\omega }_{1,i,j}$ denotes the weight of the connection between the *i*-th input neuron and the *j*-th hidden neuron, and ${b}_{1,j}$ is the bias associated with the *j*-th hidden neuron. The activation function is subsequently applied to the output of Eq. (13) to compute the output of each hidden neuron. A variety of activation functions are utilized in ML to activate neurons; among these, the sigmoid function, distinguished by its characteristic S-shaped curve, is one of the most commonly employed^34,35^. The outputs of the hidden layer neurons are calculated using Eq. (14), which mathematically defines the sigmoid function.

14 $${s}_{j}=\frac{1}{1+\text{exp}\left(-{u}_{j}\right)},j=\text{1,2},\cdots N$$

Similarly, the output of the MLP is computed by aggregating the outputs of the hidden layer neurons, the weights of the connections between the hidden layer neurons and the output layer neurons, and the biases associated with the output layer neurons, as described by Eq. (15).

15 $${y}_{j}=\frac{1}{1+\text{exp}\left(-\left(\sum_{i=1}^{N} {\omega }_{2,i,j}\cdot {s}_{i}-{b}_{2,j}\right)\right)},j=\text{1,2},\cdots O$$

Here, ${\omega }_{2,i,j}$ represents the weight of the connection from the *i*-th hidden layer neuron to the *j*-th output layer neuron, while ${b}_{2,j}$ denotes the bias associated with the *j*-th output layer neuron.

### MLP Training using LWSSA

The training process of a MLP involves iteratively refining the weights connecting the layers and the associated biases to achieve the desired outputs. This process requires meticulous fine-tuning of the weights and biases to approximate the optimal solution effectively. Proper adjustment minimizes the total error of the MLP, enhancing its predictive accuracy. However, the susceptibility of the MLP to errors arising from inadequately fine-tuned weights and biases is the focal point of this research, highlighting the importance of addressing this critical aspect to improve the network’s performance and reliability^36–38^.

**Fig. 4:**
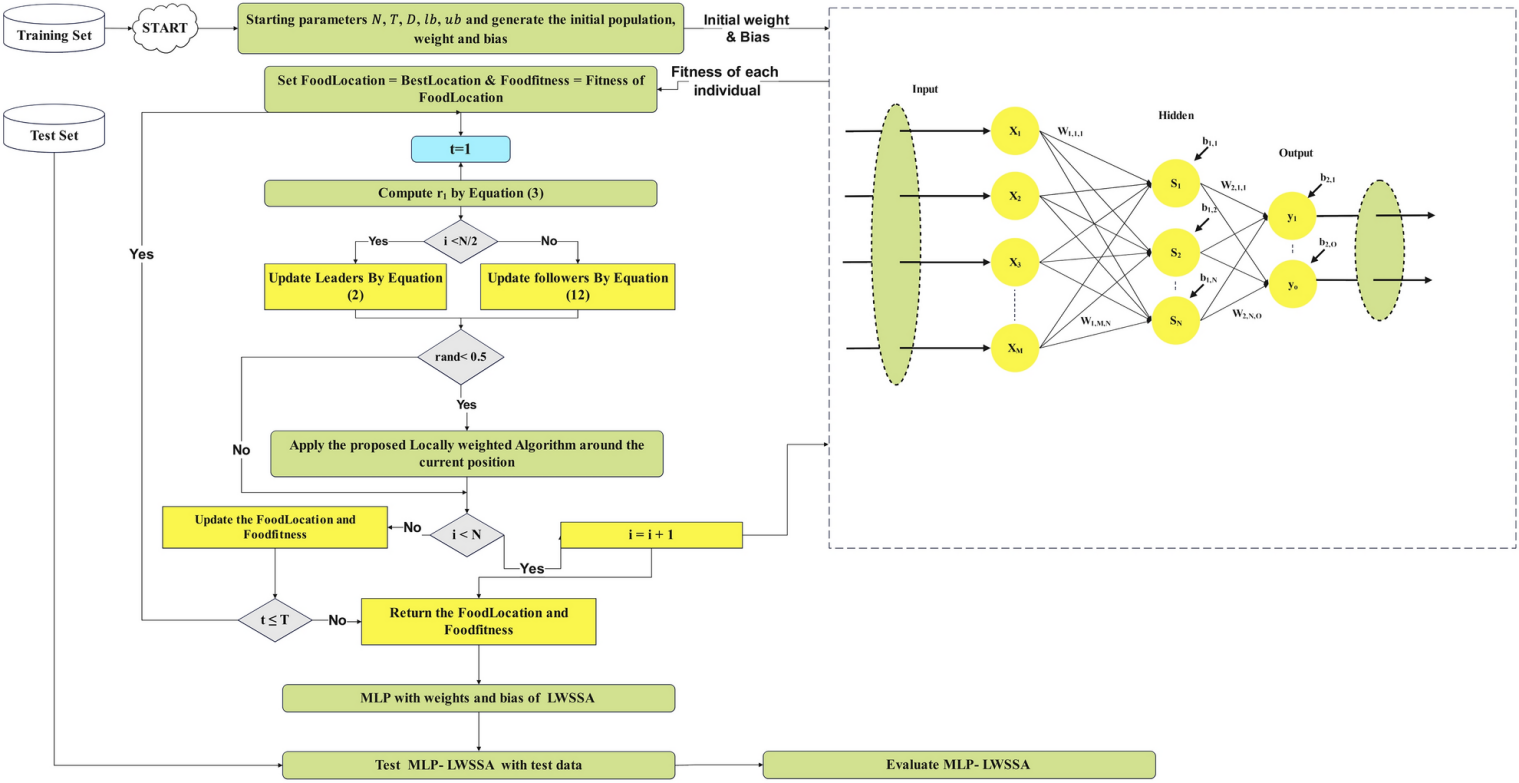
LWSSA-MLP Frame Work.

The training process of MLPs, commonly referred to as learning, is a highly intricate operation that significantly influences the effectiveness and capabilities of MLPs in addressing diverse problems. This process is pivotal as it establishes the MLP’s understanding of the complex relationships between input and output data. Recent advancements have introduced a widely recognized paradigm that employs state-of-the-art nature-inspired optimization algorithms to enhance the tuning of bias and weight values in MLP neurons. However, despite its potential, this paradigm is not without challenges, necessitating further refinement to overcome associated limitations and achieve optimal performance^33^. The representational challenge faced by the search population of an optimizer arises in defining variables of the function that mathematically models the problem and formulating the problem into a suitable objective function for the optimizer. To address this, it is crucial to represent the linking biases and weights between neurons in a manner that ensures effective communication between the optimizer’s population and enables efficient exploration of the problem space. In the LWSSA-MLP framework, the variables of the objective function, namely biases, and weights, are represented sequentially as vectors. This sequential arrangement adheres to the structure of the MLP, beginning at the input layer and concluding at the output layer. The search process performed by salps in the LWSSA for MLP optimization, as illustrated in Fig. 4, is mathematically described by Eq. (16).

16 $$l=\left[\begin{array}{llll}{\omega }_{\text{1,1},1}\cdots {\omega }_{1,M,N}& {\omega }_{\text{2,1},1}\cdots {\omega }_{2,N,O}& {b}_{\text{1,1}}\cdots {b}_{1,\text{N}}& {b}_{\text{2,1}}\cdots {b}_{2,\text{O}}\end{array}\right]$$

The dimensionality of the vector representing each search salp in the LWSSA can be calculated using the formulation provided in Eq. (17). This equation ensures that the representation aligns with the number of weights and biases in the MLP, capturing all necessary parameters for effective optimization.

17 $$D=M\cdot N+N\cdot O+N+O$$

The objective function, often referred to as the fitness function, is defined as the MSE in the LWSSA-MLP model. To calculate the MSE, the MLP is trained using a designated set of training data. The objective function evaluates the discrepancy between the outputs predicted by the MLP and the actual target outputs, as expressed in Eq. (18). This measure serves as the basis for optimizing the MLP by minimizing the error, thereby improving its accuracy and performance.

18 $$MSE^{ \circ } = \sum\nolimits_{(j = 1)}^{O} {(y^{\prime}_{j} - y_{j} )^{2} }$$

$y^{\prime}_{j}$ represents the actual target value, while ${y}_{j}$ denotes the predicted output generated by the LWSSA-MLP model for the *j*-th output neuron. The objective function, which quantifies the MSE across all predictions, is formally expressed in Eq. (19). Here, $T$ denotes the total number of samples within the training dataset, serving as the basis for evaluating and minimizing the model’s error.

19 $$F(l)=\frac{\sum_{k=1}^{Z} {\text{MSE}}_{k}}{T}$$

The training process of an MLP can be formulated as a standard optimization problem. Specifically, it involves minimizing the value of the objective function, as defined in Eq. (20). This formulation enables the application of advanced optimization techniques to effectively fine-tune the weights and biases of the MLP, thereby improving its performance.

20 $$\text{minimize : }F(l)$$

Figure 4 illustrates the application of the LWSSA optimizer in training MLP. Each salp within the LWSSA framework represents the connecting biases and weights in the MLP architecture. The MSE of the MLP across all training samples is used as the fitness value for the corresponding salp. The LWSSA employs an iterative approach to update the positions of the salps, using Eqs. (2), (12), and (9), where each position represents a potential solution to the optimization problem of identifying the optimal biases and weights for the MLP. Through this iterative process, the LWSSA minimizes the MSE over the training dataset, progressively refining the salps’ positions to enhance the performance of the MLP.

## Experimental results and analysis

To validate the efficacy of the proposed LWSSA, this subsection compares its optimization capabilities against existing nature-inspired algorithms using the CEC2015 benchmark problems. These benchmark problems encompass a diverse range of properties, including single-modal functions, multi-modal functions, hybrid functions, and composite functions^39^. Detailed descriptions and optimal solutions for the CEC 2015 benchmark problems are expressed in^39^. The test problems within the CEC2015 benchmark are categorized based on their characteristics: F1-F2 represent single-modal problems, F3-F5 correspond to multi-modal challenges, F6-F8 are hybridized problems, and F9-F15 are composite functions. The comparative analysis is conducted using statistical measures such as the mean and standard deviation of the solutions achieved by each optimizer for these problems. To reduce the influence of randomness, the results are obtained over 30 independent runs for each problem. Each run comprises 2000 iterations with a population size of 30 individuals for all compared algorithms, ensuring a robust and reliable evaluation. The contrasted Optimization algorithms include Exponential Distribution Optimizer (EDO)^40^, Moth Flame Optimization (MFO)^41^, Salp Swarm Algorithm (SSA)^31^, Transient Search Optimization (TSO)^42^, Young’s Double-Slit Experiment Optimizer (YDSE)^43^, Honey Badger Algorithm (HBA)^44^, Random Walk Grey Wolf Optimizer (RWGWO)^45^, Gold Rush Optimizer (GRO)^46^, and African Vultures Optimization Algorithm (AVOA)^47^ the parameters settings of each algorithm is given in Table 1.

**Table 1:** Parameters of Compared Optimizers.

Algorithms	Parameter setting
EDO	Switch Parameter = 0.5
MFO	b = 1
SSA	$\text{c}1= [2/e,2]$
TSO	$k=2$,$z\in [\text{0,2}] k=2$
YDSE	$\lambda =5\times {10}^{-6}$, $d=5\times {10}^{-3},$ $\delta =0.38$,$L=1, I=0.01$
SCA	$a$ $=2$
MFO	b = 1, a = [-2, -1]
HBA	$\beta =6$, C = 2
RWGWO	${a}_{0}=2$
GRO	${l}_{1}=1$, ${l}_{2}=2$
AVOA	L_1_ = 0.8, L_2_ = 0.2, *w* = 2.5, P_1_ = 0.6, P_2_ = 0.4, P_3_ = 0.6

### Benchmark analysis of the proposed LWSSA on CEC2015

#### Statistical and Non-Parametric Comparison of LWSSA and Other Optimizers

The numerical results obtained by applying metaheuristic optimizers to 15 benchmark test problems from the CEC2015 suite are presented in Table 2. The table provides the average performance and variance of the optimizers for each problem. Problems F1-F2, being single-modal, have a single global optimum solution. These problems are well-suited for evaluating an optimizer’s ability to converge precisely to the global solution. In contrast, F3-F5 are multi-modal problems characterized by multiple optimal solutions. These problems assess the optimizer’s capability to traverse a vast solution space while avoiding premature convergence to local optima. This evaluation highlights the algorithm’s ability to effectively balance exploration and exploitation. The hybridized problems, F6-F8, are designed to test an optimizer’s capacity to explore diverse regions of the problem space and intensively refine potential solution areas. Although these problems often feature a single globally acceptable solution, the surrounding terrain is typically complex, with substantial variations and numerous local optima resulting from the combination of different problem categories. Composite problems, F9-F15, integrate multiple distinct functions into a unified function. These problems are intricately constructed to evaluate an algorithm’s ability to optimize across several distinct problem characteristics simultaneously. While composite problems generally have a single global optimum, the highly complex and varied landscapes they present, replete with numerous suboptimal regions, pose significant challenges in identifying the best solution.

**Table 2:** Results of Optimizers on CEC2015 Test Suite.

		EDO	MFO	SSA	TSO	YDSE	RWGWO	AVOA	GRO	HBA	LWSSA
F1	AVG	1.375E + 10	7.991E + 9	7.028E + 3	6.951E + 10	6.890E + 10	7.978E + 5	2.852E + 8	1.747E + 4	4.381E + 5	**1.043E + 3**
	SD	6.303E + 9	5.023E + 9	4.972E + 3	6.993E + 9	7.464E + 9	6.101E + 5	2.219E + 8	3.047E + 4	6.528E + 5	**1.033E + 3**
F2	AVG	3.916E + 4	9.478E + 4	1.414E + 4	6.176E + 4	1.385E + 5	1.840E + 4	5.332E + 4	2.599E + 4	3.128E + 4	**2.836E + 3**
	SD	6.457E + 3	3.252E + 4	5.752E + 3	1.770E + 3	3.290E + 4	5.105E + 3	3.262E + 3	5.248E + 3	8.736E + 3	**1.014E + 3**
F3	AVG	3.346E + 2	3.274E + 2	3.201E + 2	3.476E + 2	3.436E + 2	3.149E + 2	3.335E + 2	**3.098E + 2**	3.282E + 2	3.196E + 2
	SD	2.622	3.859	4.906	1.781	**1.380**	4.522	3.053	4.214	3.887	4.032
F4	AVG	7.920E + 3	5.366E + 3	4.073E + 3	9.592E + 3	8.759E + 3	**3.500E + 3**	5.290E + 3	4.157E + 3	7.821E + 3	3.804E + 3
	SD	4.265E + 2	7.906E + 2	8.329E + 2	**3.356E + 2**	4.045E + 2	6.234E + 2	5.402E + 2	4.940E + 2	6.183E + 2	4.736E + 2
F5	AVG	5.037E + 2	5.011E + 2	5.004E + 2	5.063E + 2	5.042E + 2	**5.003E + 2**	5.016E + 2	5.009E + 2	5.031E + 2	**5.003E + 2**
	SD	7.163E-1	5.519E-1	2.285E-1	1.015	4.256E-1	1.342E-1	7.871E-1	3.187E-1	4.689E-1	**1.289E-1**
F6	AVG	6.026E + 2	6.013E + 2	6.006E + 2	6.056E + 2	6.061E + 2	6.004E + 2	6.005E + 2	**6.003E + 2**	6.004E + 2	6.004E + 2
	SD	1.030	8.860E-1	1.721E-1	2.840E-1	4.118E-1	1.090E-1	9.582E-2	**7.065E-2**	1.003E-1	8.070E-2
F7	AVG	7.326E + 2	7.159E + 2	7.007E + 2	8.162E + 2	8.525E + 2	7.004E + 2	7.004E + 2	7.003E + 2	7.006E + 2	**7.002E + 2**
	SD	1.077E + 1	1.613E + 1	3.531E-1	7.462	1.795E + 1	2.475E-1	1.593E-1	7.627E-2	2.626E-1	**2.805E-2**
F8	AVG	8.424E + 4	2.359E + 5	8.099E + 2	3.663E + 7	3.513E + 7	8.091E + 2	1.321E + 3	**8.072E + 2**	8.193E + 2	8.096E + 2
	SD	8.031E + 4	1.295E + 5	4.845	1.247E + 7	1.537E + 7	2.138	7.410E + 2	**1.270**	7.601	3.740
F9	AVG	9.137E + 2	9.133E + 2	9.124E + 2	9.138E + 2	9.139E + 2	**9.114E + 2**	9.130E + 2	**9.114E + 2**	9.127E + 2	9.124E + 2
	SD	1.789E-1	3.669E-1	4.810E-1	**1.235E-1**	1.858E-1	5.935E-1	3.850E-1	4.713E-1	6.747E-1	4.223E-1
F10	AVG	4.364E + 6	9.660E + 5	4.756E + 5	2.276E + 8	8.850E + 7	4.824E + 5	8.931E + 6	6.232E + 5	3.796E + 5	**2.048E + 4**
	SD	3.800E + 6	6.626E + 5	3.437E + 5	1.131E + 8	3.129E + 7	2.901E + 5	5.125E + 6	3.793E + 5	3.617E + 5	**1.082E + 4**
F11	AVG	1.197E + 6	4.023E + 3	3.430E + 3	3.036E + 8	9.856E + 8	3.631E + 3	5.663E + 4	2.097E + 3	2.719E + 3	**1.138E + 3**
	SD	1.263E + 6	2.157E + 3	3.271E + 3	7.062E + 7	5.618E + 8	3.590E + 3	7.595E + 4	2.015E + 3	2.564E + 3	**1.034E + 2**
F12	AVG	1.097E + 8	3.637E + 3	2.226E + 3	9.139E + 12	3.696E + 13	2.772E + 3	9.236E + 12	3.837E + 3	3.540E + 3	**1.382E + 3**
	SD	1.877E + 8	1.744E + 3	4.502E + 2	4.012E + 12	2.113E + 13	6.832E + 2	4.682E + 12	7.583E + 2	9.737E + 2	**1.104E + 2**
F13	AVG	1.738E + 3	1.629E + 3	1.577E + 3	3.583E + 3	2.337E + 3	1.558E + 3	1.639E + 3	1.558E + 3	1.559E + 3	**1.548E + 3**
	SD	6.167E + 1	4.751E + 1	1.897E + 1	7.188E + 2	2.082E + 2	6.217	3.639E + 1	6.217	**6.324E-2**	1.576E + 1
F14	AVG	6.026E + 3	2.014E + 3	2.105E + 3	1.106E + 4	1.004E + 4	2.029E + 3	5.598E + 3	**1.972E + 3**	2.066E + 3	2.070E + 3
	SD	6E + 2	6.299E + 1	8.723E + 1	3.151E + 3	9.881E + 2	6.125E + 1	3.937E + 3	**5.026E-2**	1.003E + 2	1.051E + 2
F15	AVG	2.829E + 3	2.621E + 3	2.320E + 3	3.074E + 03	3.235E + 3	2.683E + 3	2.829E + 3	2.617E + 3	2.890E + 3	**1.940E + 3**
	SD	3.383E + 2	1.246E + 2	9.614E + 1	**1.873E + 01**	7.908E + 1	1.183E + 2	8.404E + 1	1.250E + 2	3.376E + 1	1.117E + 2
		**EDO**	**MFO**	**SSA**	**TSO**	**YDSE**	**RWGWO**	**AVOA**	**GRO**	**HBA**	**LWSSA**
	FV	7.5111	5.6578	3.5667	9.3333	9.4333	3.289	6.3156	2.9733	4.8089	**2.111**
	FR	8	6	4	9	10	3	7	2	5	**1**
	P-Value	2.799e-7	2.134e-6	5.408e-6	2.314e-7	1.346e-3	1.945e-7	5.8296e-7	7.326e-7	5.472e-3	-

Significant values are in bold.

This statistical analysis provides a comprehensive evaluation of an optimizer’s performance across a diverse range of problem types, highlighting its versatility and robustness in addressing various optimization challenges. The efficiency of the proposed LWSSA algorithm was assessed and compared with existing comparable techniques using the CEC2015 benchmark suite, as detailed in Table 2. The problems involve a dimensionality of 30 decision variables. Notably, lower mean values indicate higher efficiency, while lower variance signifies greater stability in consistently achieving optimal solutions. The best-performing results are highlighted in bold for clarity. The findings in Table 2 clearly illustrate the advantages of the LWSSA. Specifically, the LWSSA outperformed other optimizers on basic functions F1 and F2, which are single-modal in nature. For multi-modal problems, the LWSSA achieved the most optimal average solution on function F5, performing comparably to the improved RWGWO. In hybrid functions, the LWSSA demonstrated superior performance on function F7, surpassing other algorithms in its ability to balance exploration and exploitation. In composite problems, the LWSSA’s performance was slightly higher than RWGWO and GRO on F9, while the GRO algorithm obtained the most optimal solution for F14. Nonetheless, the LWSSA algorithm achieved the best average solutions on functions F10, F11, F12, F13, and F15. Additionally, the LWSSA exhibited lower variance across the test problems, indicating its robustness and reliability compared to other optimizers. The results demonstrate that the LWSSA outperforms other algorithms across a wide array of benchmark problems. The enhancements implemented in the LWSSA, including the incorporation of the LWM, have proven effective in refining solutions through efficient local search. Furthermore, the newly introduced MM has significantly enhanced the algorithm’s global search capabilities. Together, these improvements enable the LWSSA to attain superior solutions, making it a highly competitive optimizer for complex problem-solving scenarios.

The efficiency of the proposed LWSSA was further validated using Friedman’s non-parametric test in comparison with various optimization approaches. Table 2 presents the mean ranks of all optimizers based on their average performance across the CEC2015 benchmark problems. The results clearly demonstrate that LWSSA, ranked first, attaining a mean rank of 2.111 and securing the first position among the evaluated optimizers. Additionally, the Wilcoxon Rank Sum test was employed to assess whether there were statistically significant differences in efficiency between LWSSA and the other algorithms, using a significance threshold of 0.05. H0 represents the null hypothesis, which assumes that the performance of all competing optimizers is equivalent, while H1 represents the alternative hypothesis, suggesting that a significant improvement exists in the results of the compared optimizer if the p-value is less than or equal to 0.05. Referring to Table 2, it is evident that the p-value for LWSSA, when compared to the other algorithms, is less than 0.05. This result indicates statistically significant distinctions in performance, confirming that LWSSA outperforms the other algorithms with a measurable enhancement in efficiency.

#### Convergence analysis

**Fig. 5:**
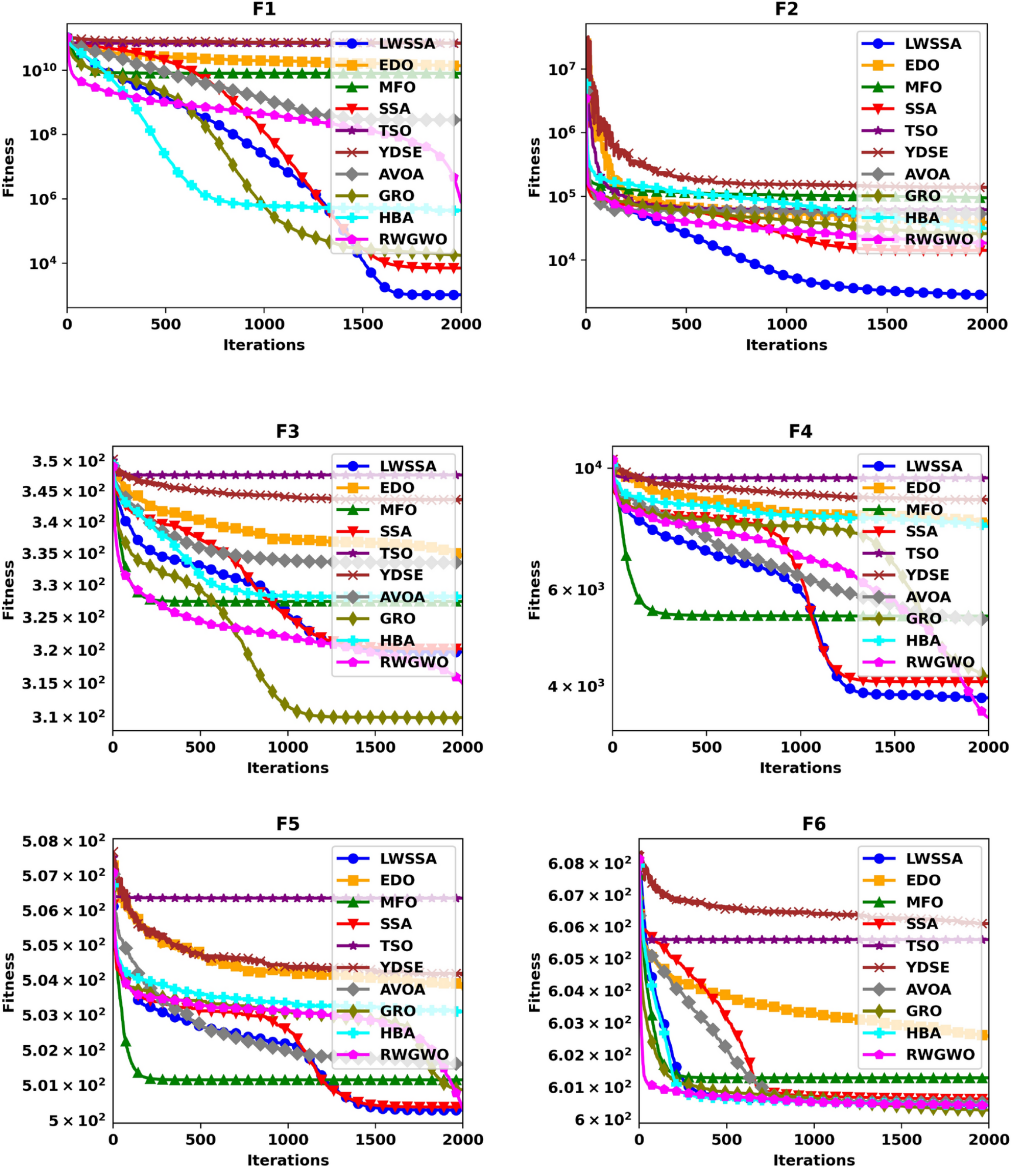

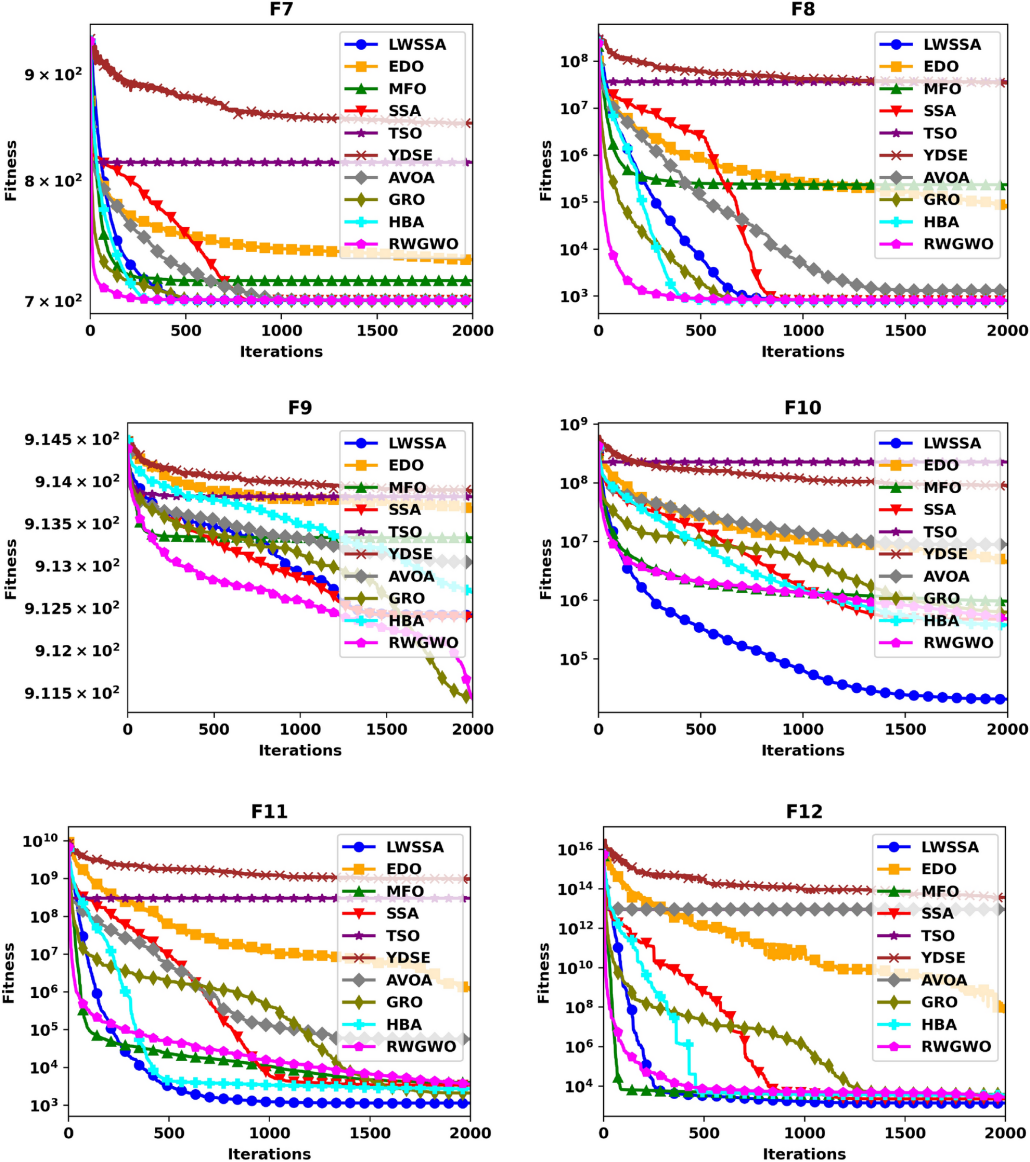

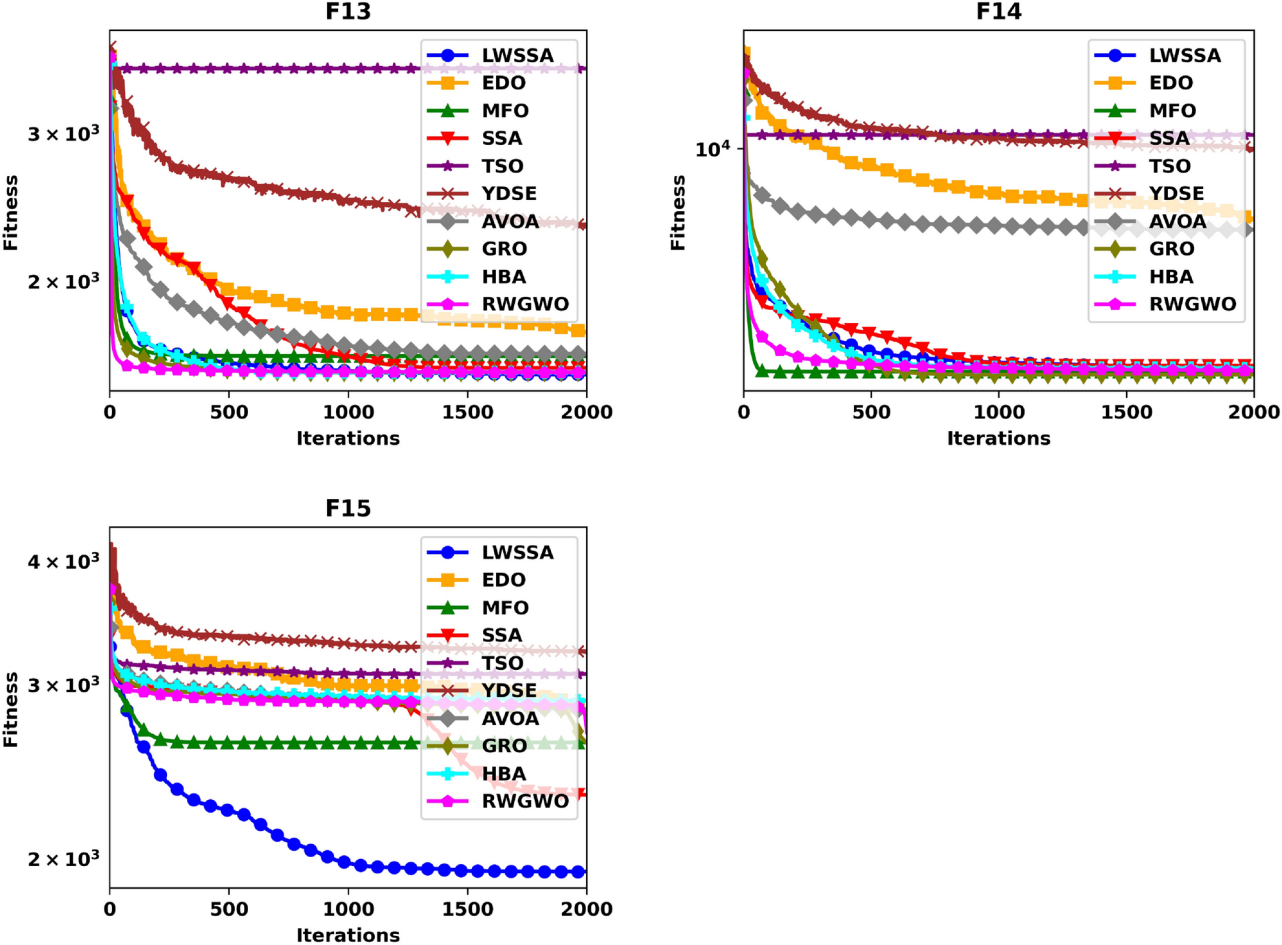
Convergence Graphs of LWSSA and Compared Optimizers.

Figure 5 illustrates the convergence plot of ten optimization methods, offering a comparative evaluation of their effectiveness in achieving convergence when addressing optimization problems. The problem dimension for functions F1-F15 is set to 30. The superiority of LWSSA in terms of convergence accuracy is particularly evident in its application to the single-modal problems F1 and F2. For multi-modal problems such as F5, LWSSA demonstrated an exceptional rate of convergence, achieving near-optimal solutions with remarkable efficiency. In the hybridized problem F7, the LWSSA exhibited a slightly higher convergence level compared to the standard SSA and significantly outperformed other algorithms in terms of both convergence speed and accuracy. Similarly, in composite problems F10, F11, F12, F13, and F15, the LWSSA consistently achieved superior convergence trajectories, clearly outperforming the standard SSA and other comparative methods. The convergence plots presented in Fig. 5 highlight the efficacy of the enhancements implemented in LWSSA, particularly in its ability to balance exploration and exploitation, leading to faster and more accurate optimization outcomes.

These findings demonstrate the enhanced capacity of LWSSA to efficiently optimize complex problems and achieve convergence to near-optimal solutions. Notably, the rapid convergence of the LWSSA method underscores its suitability for optimization tasks where swift convergence is critical, such as in the optimization of MLP parameters. This capability makes LWSSA a highly effective and reliable tool for addressing optimization challenges where time and accuracy are paramount.

#### Box Plot analysis

**Fig. 6:**
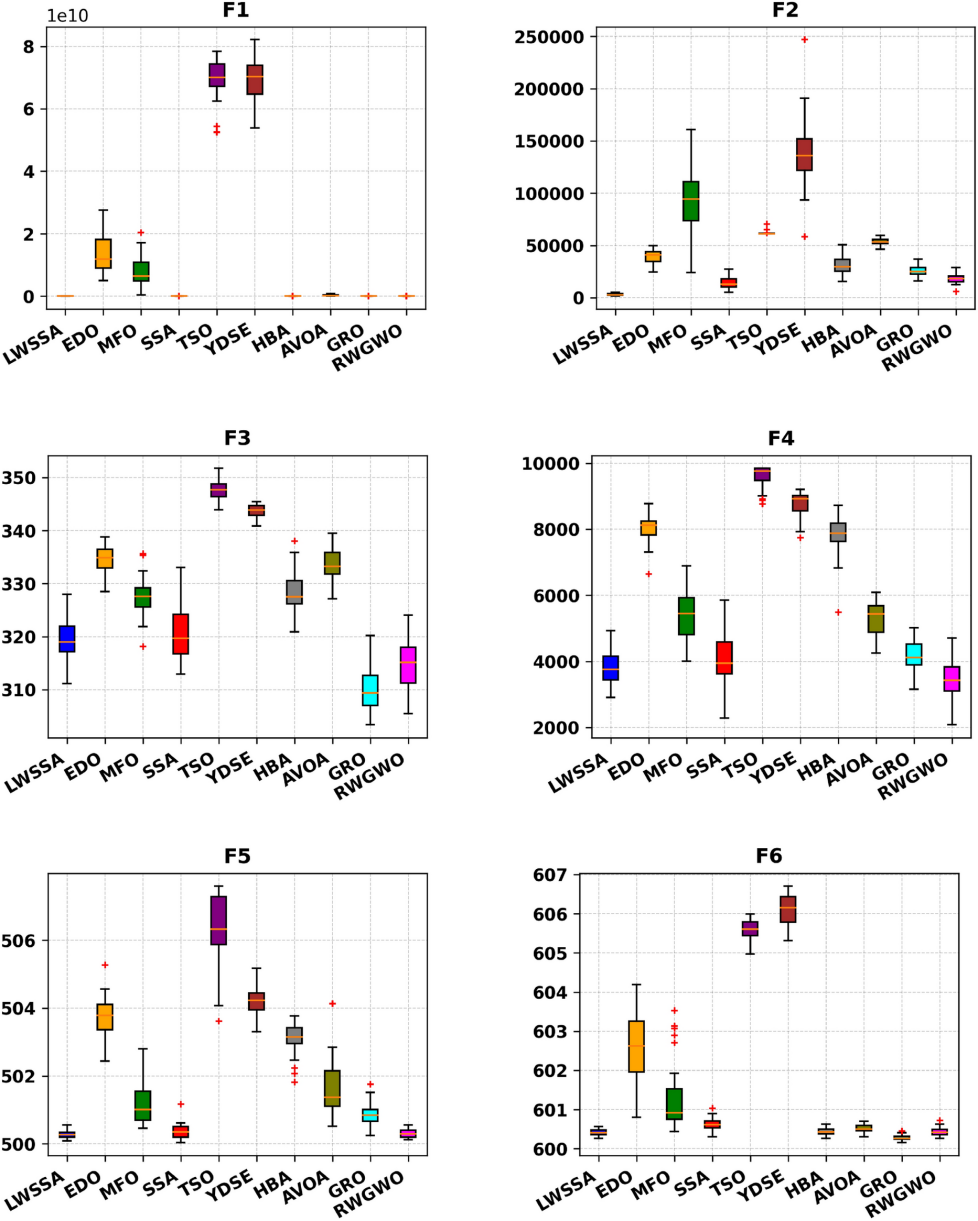

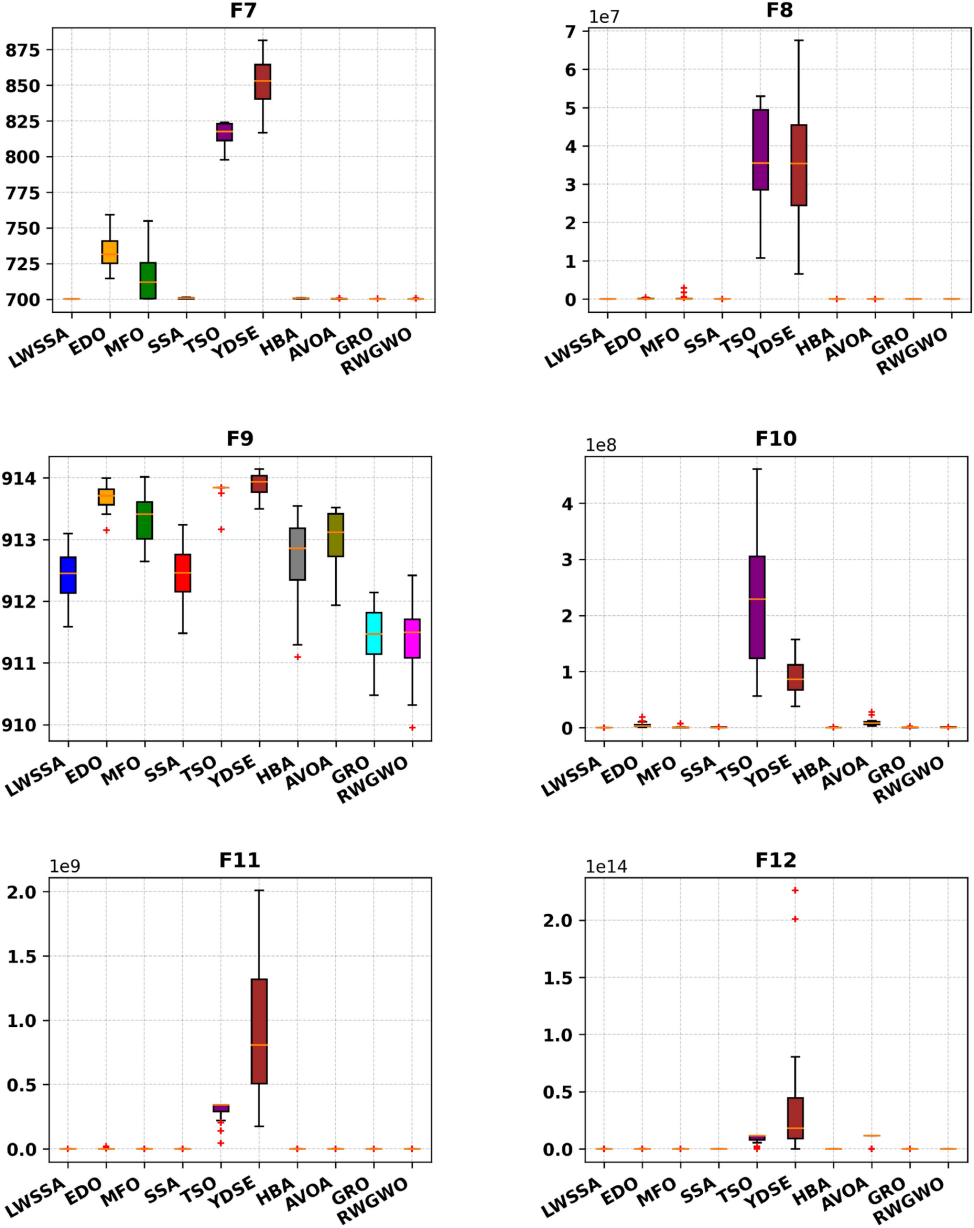

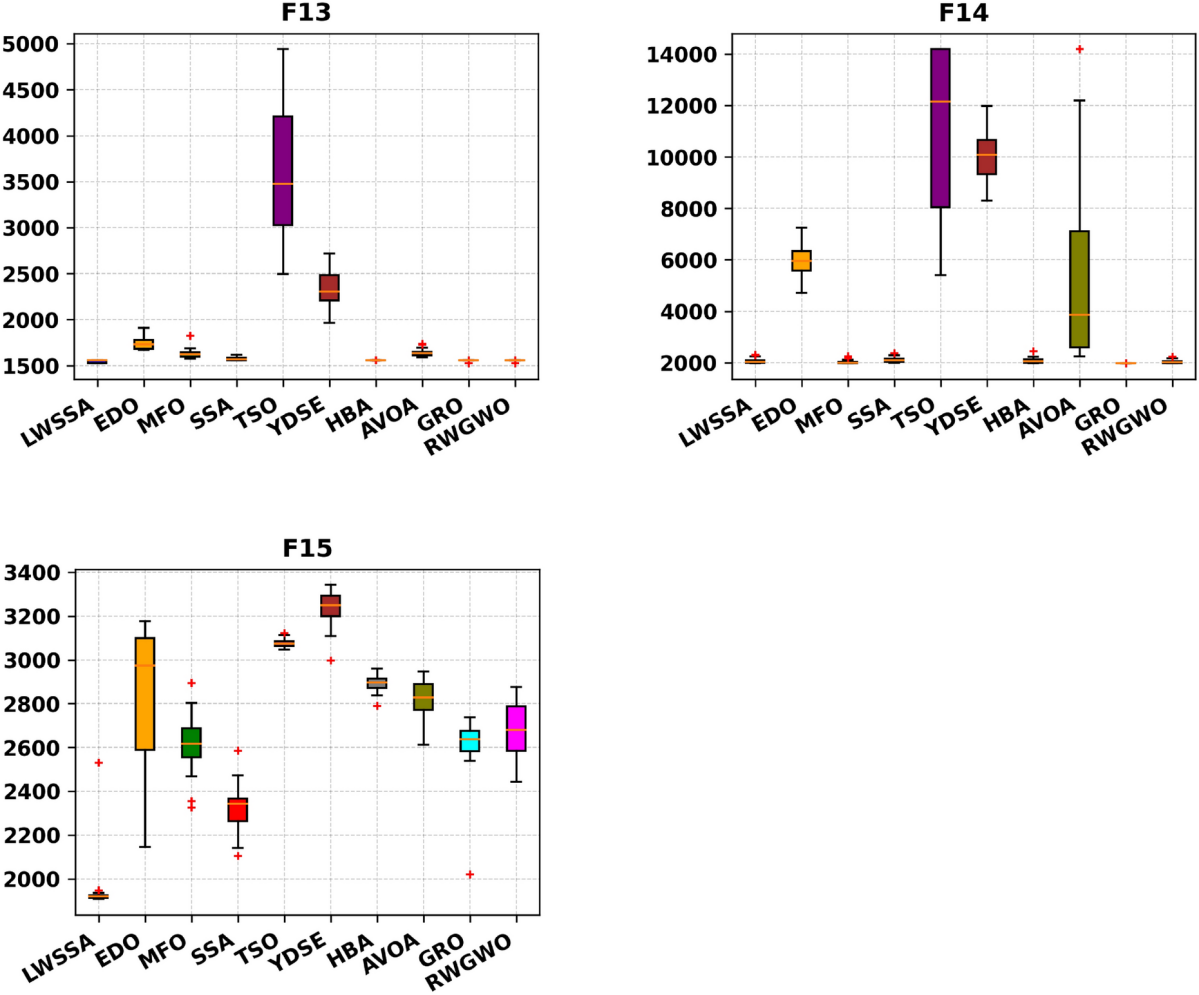
Box Plot of LWSSA and Compared Optimizers on CEC2015 Functions.

The box plot visualizations in Fig. 6 provide a concise representation of the distribution of the most optimal outcomes for the LWSSA algorithm compared to its counterparts on benchmark problems. Each box plot highlights key statistical measures such as the mean, which is represented by the central marker, while the box edges denote the 75th and 25th percentiles. The whiskers extend to illustrate the range of the data, excluding outliers, which are depicted separately as red " + " symbols. Each box plot captures the optimal solution obtained from 30 independent repetitions. The box plots for functions F1-F15 reveal that the LWSSA generally exhibits a narrow interquartile range (IQR). This characteristic suggests that the LWSSA consistently achieves results close to the average value across multiple iterations. The small IQR further indicates that LWSSA effectively identifies solutions near the optimal result, with minimal variation among iterations, highlighting the consistency and reliability of its search procedure.

Although box plots do not explicitly illustrate the convergence rate, the dispersion and bias of the data can be inferred from the plot structure. For functions F1, F2, F7, F8, F10, F11, F12, and F13, the concentration of LWSSA outcomes toward the lower end of the scale reflects consistent convergence toward the optimal solution. However, in certain cases, such as F9, the LWSSA demonstrates a comparatively wider IQR, though it remains narrower than those of other optimizers like HBA and GRO. This suggests that while LWSSA maintains robust performance, it may exhibit reduced resilience in addressing specific areas of the problem space for such cases. In summary, the LWSSA demonstrates promising performance across a variety of benchmark problems, particularly when the problem characteristics align with its optimization strategies. Its adaptability is evident from its consistently narrow IQR in most cases, which underscores its efficiency in converging to optimal solutions. Nevertheless, variations in performance across certain problem types indicate areas for potential refinement to enhance its robustness further.

#### Exploration vs exploitation analysis

Exploration refers to an optimizer’s ability to investigate diverse solutions within unexplored regions of the search space, whereas exploitation pertains to its capacity to refine solutions near the optimal result for a given problem. The F1 evaluation problems, being unimodal, are particularly well-suited for assessing an optimizer’s ability to intensify its search around promising regions. Conversely, F5 represents multi-peaked evaluation functions with numerous local optima, making it an ideal benchmark for evaluating an optimizer’s diversification capability. Hybrid and composite problems, such as F6, F7, F10, and F15, are designed to evaluate both phases’ capabilities simultaneously. As evidenced by the results in Table 2, the LWSSA model consistently achieves superior outcomes across these various problem types. The optimizer’s ability to approximate near-optimal solutions demonstrates its effectiveness in surpassing multiple local optima. This success is attributed to a well-balanced integration of exploration and exploitation phases, allowing LWSSA to navigate complex problem spaces effectively.

**Fig. 7:**
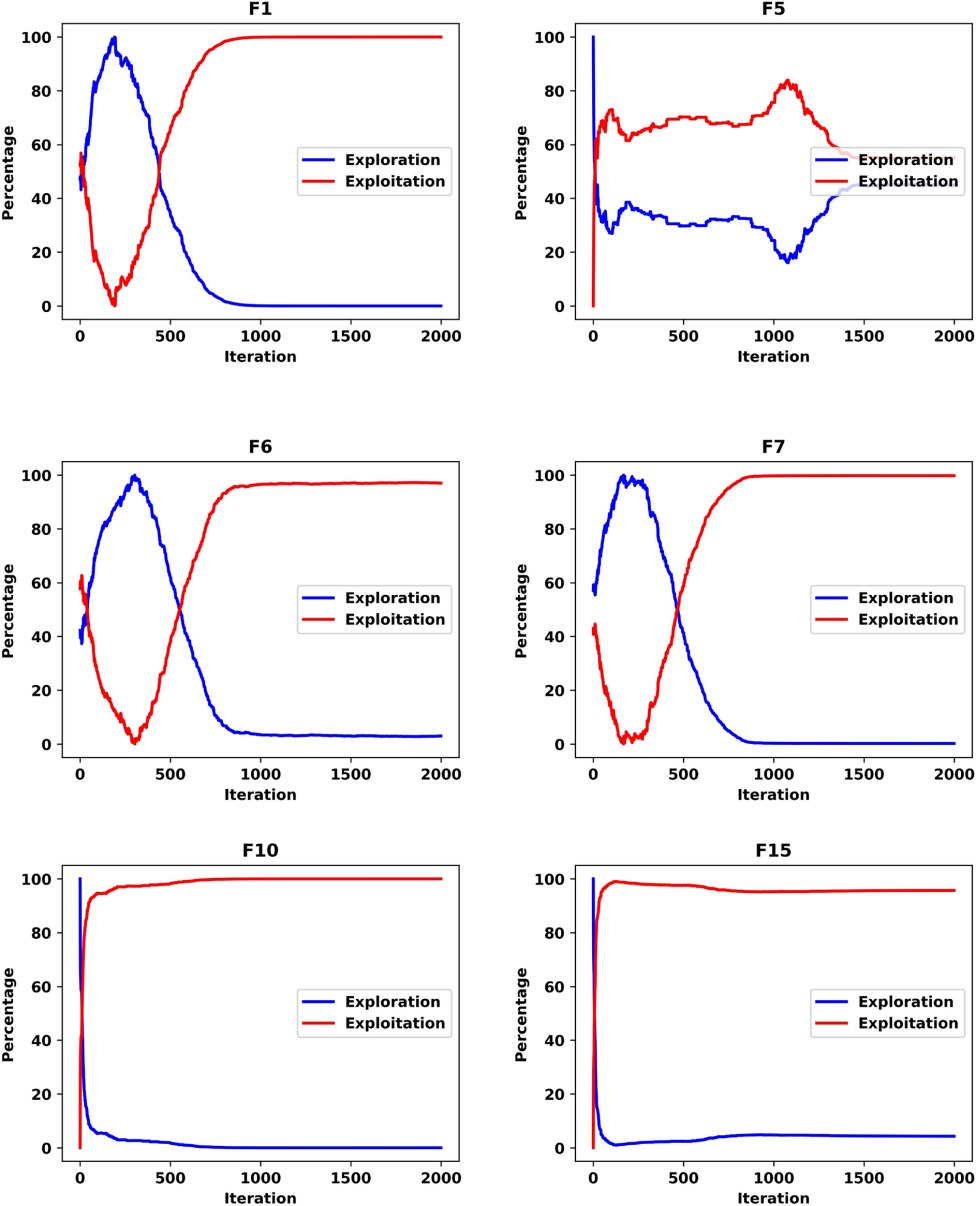
Exploitation and Exploration Graphs of LWSSA.

Figure 7 visually illustrates these two phases of the LWSSA optimizer (exploration and exploitation). The figure depicts how the optimizer initiates the search process with an extensive global exploration phase, enabling the identification of high-potential regions within the search space. Subsequently, the algorithm transitions into a focused exploitation phase, refining the solutions around promising areas. As the search progresses, the LWSSA establishes a dynamic equilibrium between exploration and exploitation, ensuring both breadth and precision in identifying optimal solutions. This balanced approach underscores the efficacy of LWSSA in addressing diverse optimization challenges.

### Carbon emission prediction analysis

#### Carbon emission dataset

This study examines key variables contributing to environmental degradation in Thailand. The datasets utilized for this investigation were sourced from reputable institutions, including the World Bank Database (WBD), the British Petroleum Database (BPD), and the KOF Swiss Economic Institute (KSEI).

**Fig. 8:**
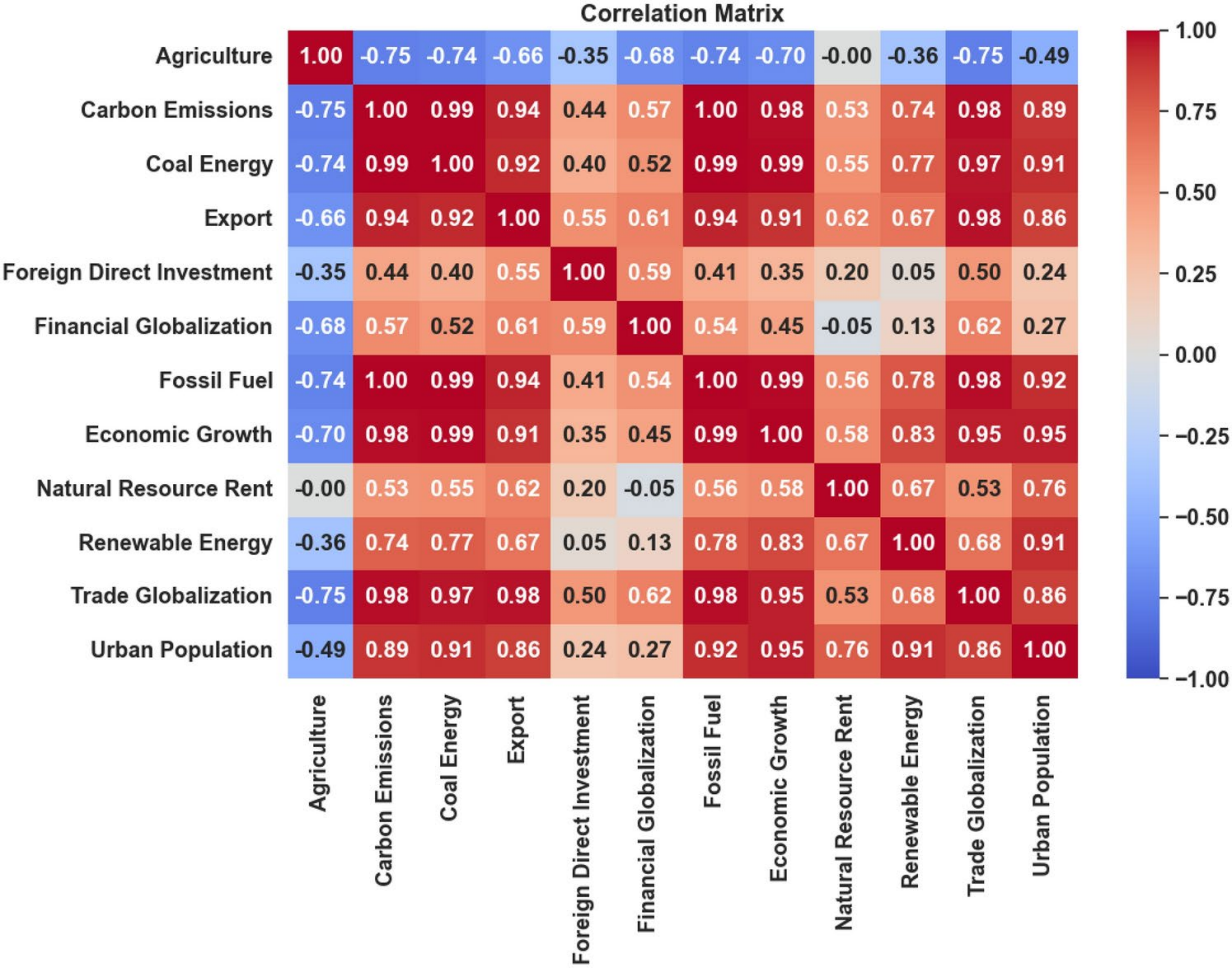
Feature Correlation Plot.

**Fig. 9:**
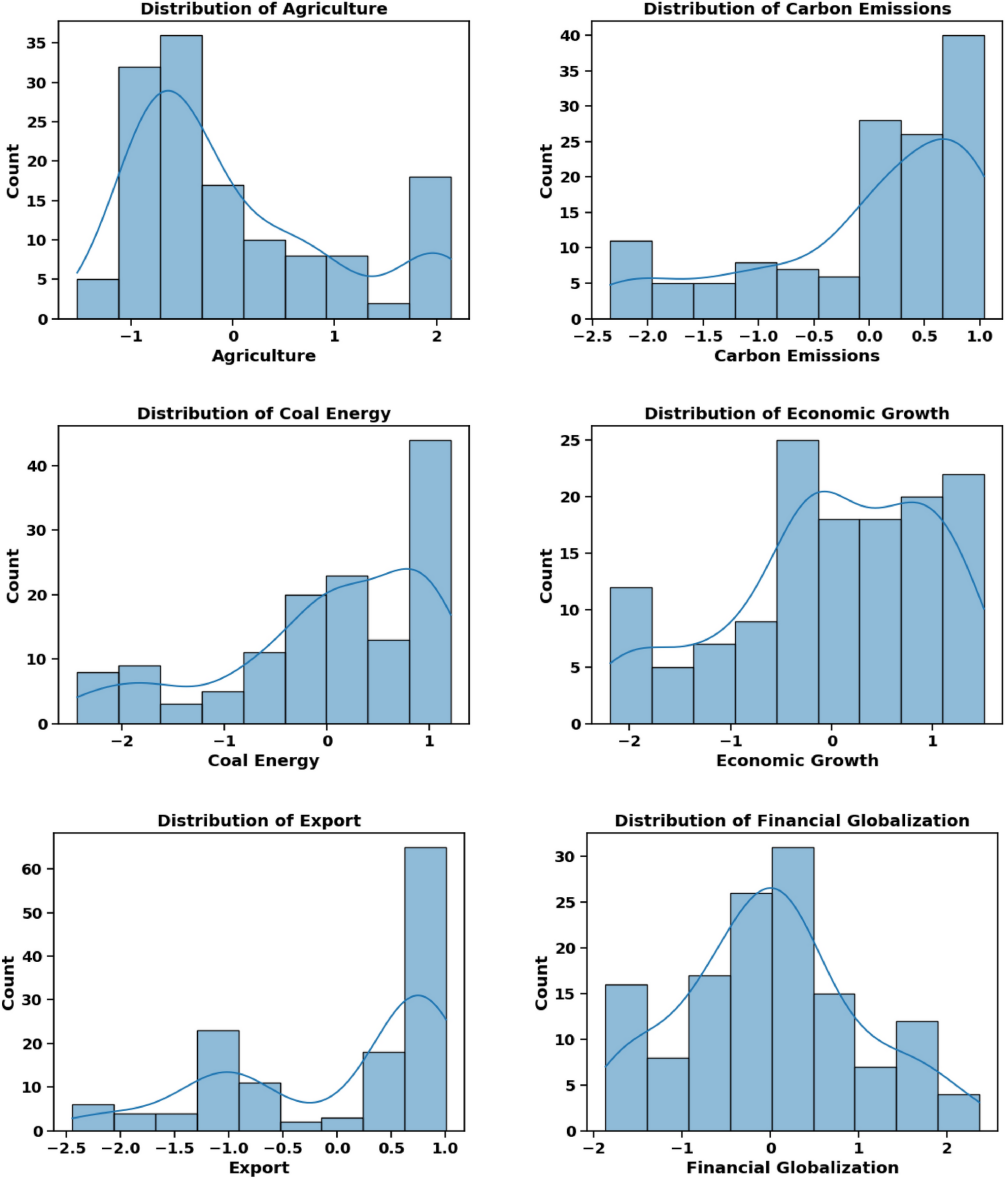

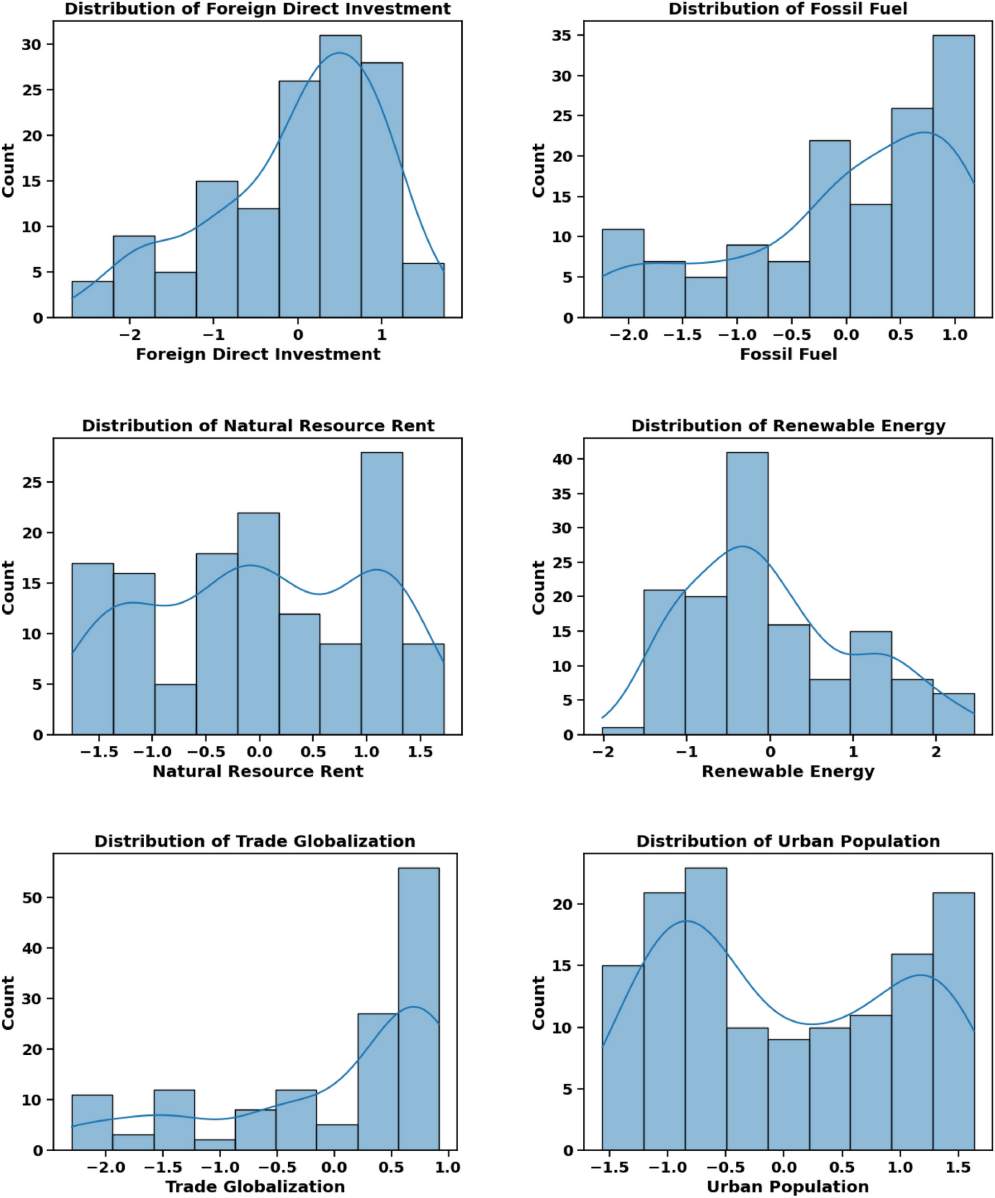
Feature Distribution.

**Fig. 10:**
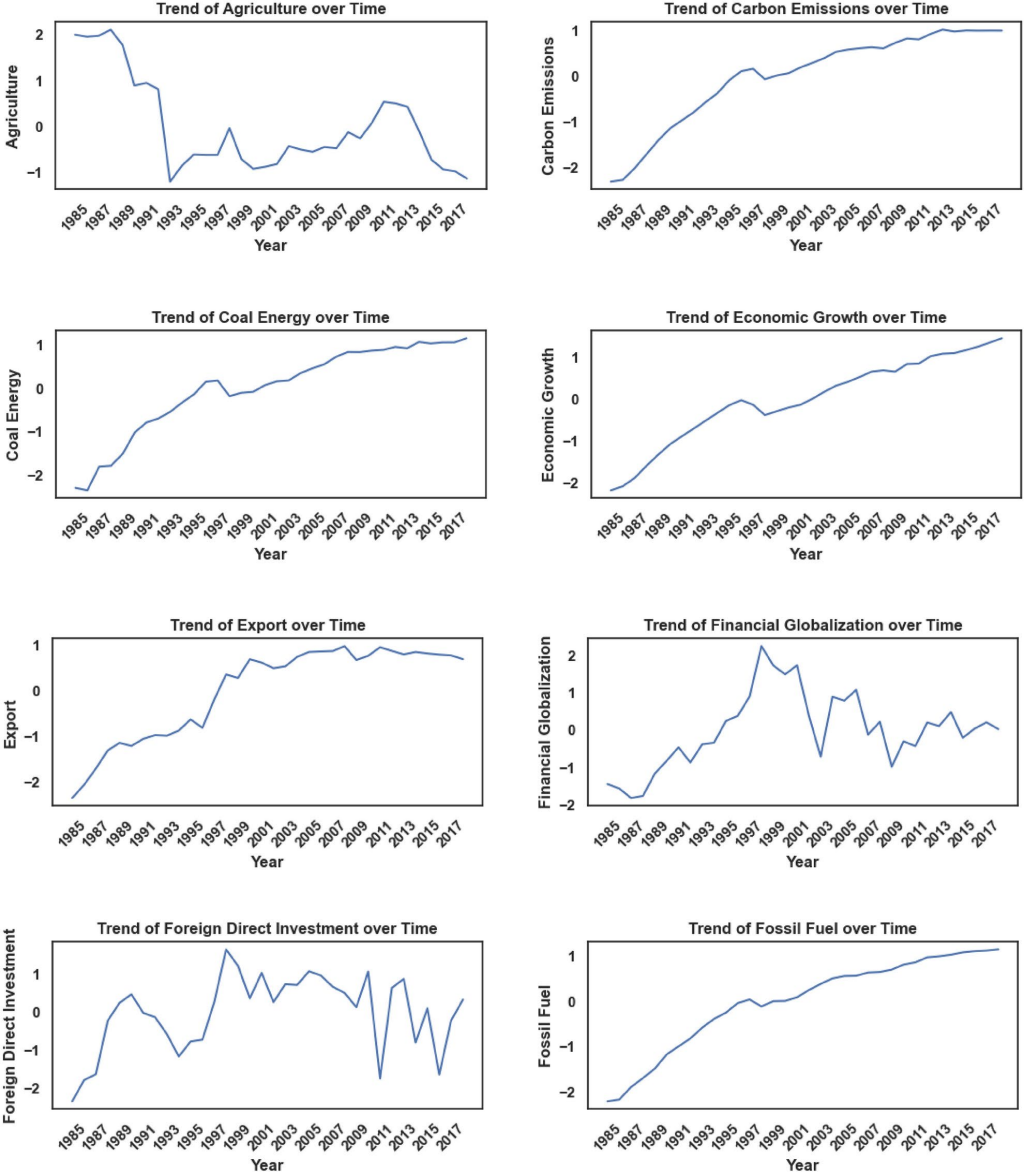

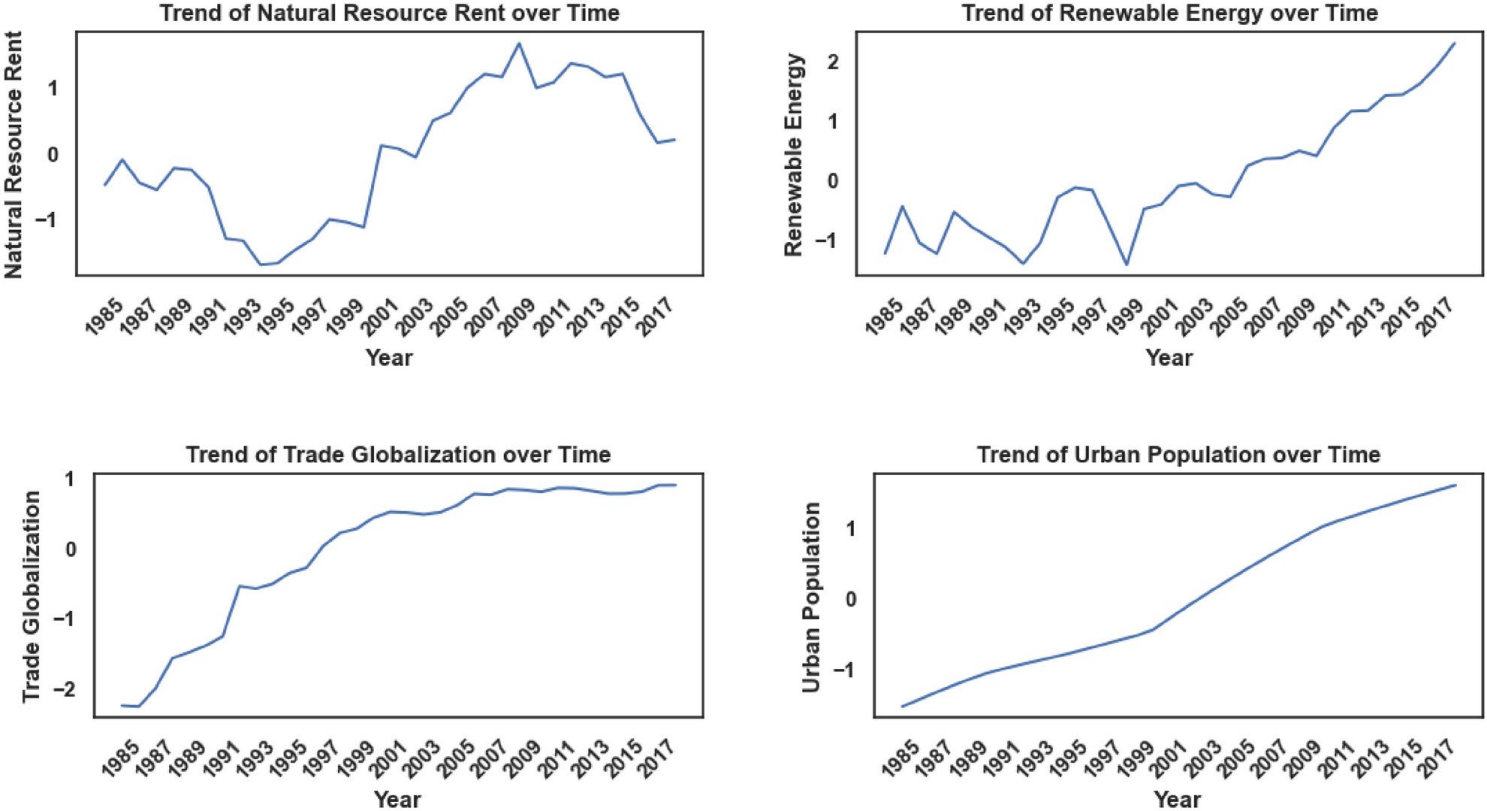
Trend of Features.

The dependent variable in this study is CO_2_ emissions, while the independent variables, referred to as input factors, include Agriculture, Coal Energy, Export, Foreign Direct Investment, Financial Globalization, Fossil Fuel, Economic Growth, Natural Resource Rent, Renewable Energy, Trade Globalization, and Urban Population. Table 3 provides a comprehensive overview of these factors. All models used in the experiment were tested and trained using quarterly data spanning from 1985 to 2018. Figures 8, 9, and 10 provide critical insights into the dataset from different perspectives. Figure 8 illustrates the correlation heatmap, highlighting the strength of relationships among variables, which is crucial for identifying key dependencies. Figure 9 shows the dispersion of each factor, revealing variability within each feature. Figure 10 presents the temporal trends of each factor, capturing their evolution over time to identify patterns, cycles, or irregularities. Together, these figures offer a comprehensive understanding of the dataset’s structure and dynamics.

**Table 3:** Characteristics of Features.

Full Name	Metrics	Source
Agriculture	% of GDP	World Bank Database
Coal Energy	TWH	British Petroleum Database
Export	% of GDP	World Bank Database
Foreign Direct Investment	% of GDP	World Bank Database
Financial Globalization	Index	KOF Swiss Economic Institute
Fossil Fuel	TWH	British Petroleum Database
Economic Growth	GDP per capita (constant 2015)	World Bank Database
Natural Resource Rent	% of GDP	World Bank Database
Renewable Energy	TWH	British Petroleum Database
Trade Globalization	Index	KOF Swiss Economic Institute
Urban Population	% of total population	World Bank Database
Carbon Emissions	Kilotonnes	World Bank Database

#### Model Performance Metrics

To assess the superiority of LWSSA in enhancing the performance of MLP, it is crucial to employ metric evaluations. These evaluations provide a comprehensive understanding of the efficacy of the LWSSA-MLP framework compared to other nature-inspired MLP models for CO_2_ emission prediction. A diverse set of error metrics and precision indicators is utilized to evaluate each technique, offering valuable insights into the reliability and accuracy of the optimized ML algorithms^19,48^. The algorithm that achieves the highest level of accuracy and precision can be identified by carefully comparing the results obtained from these performance indicators. The calculations and definitions of these performance metrics are summarized in Table 4. *N* represents the total number of data points, ${Y}_{i}^{\text{Exp}}$ refers to the *i*-th observed data point, ${Y}_{i}^{MLP}$ denotes the *i*-th predicted value any of the experimental models, and $\overline{Y }$ represents the mean of the observed data.

**Table 4:** Performance Indicators.

Metric	Formula	Definition
R^2^	$\frac{\sum_{i=1}^{N} {\left({Y}_{i}^{\text{Exp}}-{\overline{Y} }^{\text{Exp}}\right)}^{2}-\sum_{i=1}^{N} {\left({Y}_{i}^{\text{Exp}}-{Y}_{i}^{MLP}\right)}^{2}}{\sum_{i=1}^{N} {\left({Y}_{i}^{\text{Exp}}-{\overline{Y} }^{\text{Exp}}\right)}^{2}}$	Coefficient of Determination
RMSE	$\sqrt{\Sigma {\left({\text{Y}}_{\text{i}}^{MLP}-{\text{Y}}_{\text{i}}^{\text{Exp}}\right)}^{2}/\text{n}}$	Root Mean Square Error
MSLE	$\frac{1}{N}\sum_{i=0}^{N} {\left(\text{log}\left({\text{Y}}_{\text{i}}^{\text{ExP}}+1\right)-\text{log}\left({\text{Y}}_{\text{i}}^{MLP}+1\right)\right)}^{2}$	Mean Square Logarithm Error
MAE	$\frac{1}{N}{\sum }_{i=1}^{n} \left|{\text{Y}}_{\text{i}}^{\text{Exp}}-{\text{Y}}_{\text{i}}^{MLP}\right|$	Mean absolute error
MAPE	$M = \frac{1}{n}\sum\limits_{t = 1}^{n} {\left| {\frac{{Y_{i}^{Exp} - Y_{i}^{MLP} }}{{Y_{i}^{Exp} }}} \right|}$	Mean Absolute Percentage Error

#### CO_2_ Predictions Experiments, Results and Discussion

**Fig. 11:**
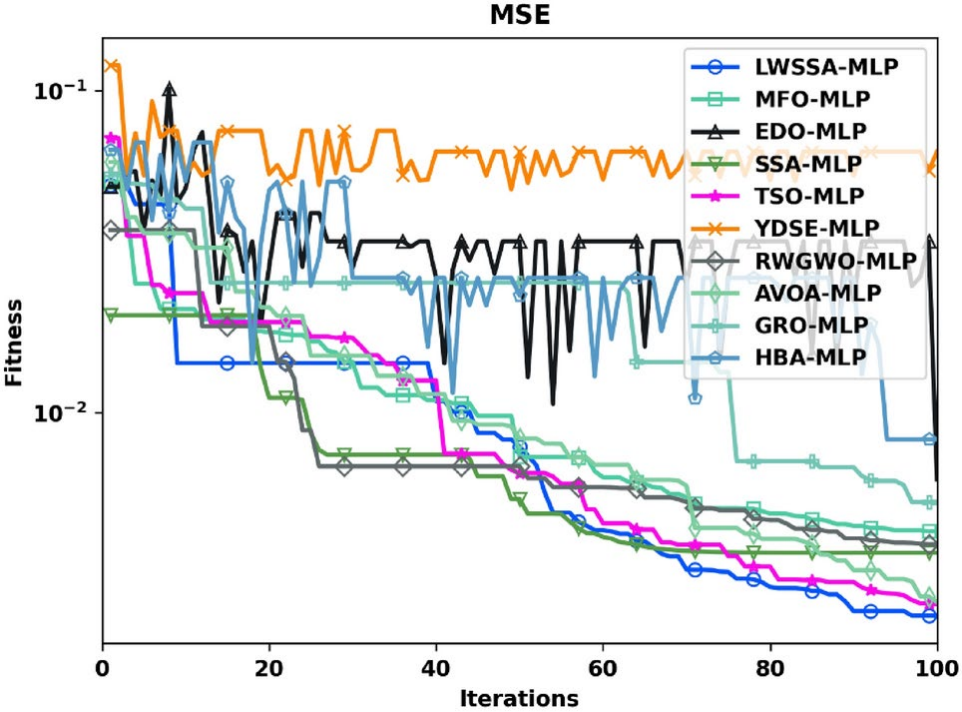
Training Phase Convergence Curve of Optimizer-based MLP Models.

The present study employs an ML-based approach to predict CO_2_ levels. To enhance the learning process of the MLP, several metaheuristic algorithms, including the newly proposed LWSSA, are evaluated. This section details the key findings of the assessment, providing an in-depth analysis and comparison of each technique’s impact on improving the MLP network’s learning efficiency. The dataset was divided into training and testing subsets using an arbitrary selection process, adhering to an 80:20 ratio. To maintain consistency across all algorithms, the search boundaries for the weights and biases of the MLP were set between -10 and 10, and the maximum iteration limit for all models is 100. Figure 11 illustrates the training phase convergence patterns of the MLP models optimized by each algorithm. After 100 iterations, the convergence rate was evaluated using the MSE as an evaluation indicator.

As shown in Fig. 11, the proposed LWSSA-MLP model achieved the best convergence performance, reaching optimal convergence within 70 iterations. This indicates that the LWSSA-MLP obtained the lowest error throughout the training process. The rapid convergence rate and low MSE of the LWSSA-MLP model is attributed to its hybrid optimization strategies, which combine the advantages of the MM and the newly introduced LWM. By effectively balancing exploitation and exploration, LWSSA-MLP achieves faster convergence to optimal solutions (minimal error during training). In comparison, the TSO-MLP model demonstrated a moderate convergence rate with a slightly lower MSE than SSA-MLP, GRO-MLP, and AVOA-MLP models. However, these models were still outperformed by the LWSSA-MLP. On the other hand, the MFO-MLP, EDO-MLP, and YDSE-MLP models exhibited significantly slower convergence rates and higher MSEs compared to the other techniques. These findings suggest that these optimization methods are less effective in balancing exploration and exploitation, potentially causing them to become trapped in suboptimal solutions. Observation shows that the LWSSA-MLP model demonstrated superior performance in terms of both convergence rate and accuracy, highlighting its efficacy in optimizing the learning process of the MLP network.

Tables 5 and 6 present the results for R^2^, RMSE, MSLE, MAE, and MAPE metrics, evaluating various optimizer-based MLP models alongside the standard MLP during both training and testing phases. A comprehensive analysis of these results demonstrates that the LWSSA-MLP framework achieves significantly lower error levels, as indicated by metrics such as RMSE, MSLE, and MAE, in comparison to peer optimizer-based MLP models. While models such as EDO-MLP, SSA-MLP, GRO-MLP, and RWGWO-MLP exhibit slightly reduced error rates, their performance remains inferior to that of the proposed LWSSA-MLP. Additionally, the R^2^ metric, which evaluates prediction precision, underscores the superior performance of the LWSSA-MLP model. The higher R^2^ value associated with LWSSA-MLP signifies exceptional capability in capturing the relationship between observed and predicted values, as evidenced in Tables 5 and 6. Notably, the R^2^ score for LWSSA-MLP in predicting CO₂ levels using previously unseen data is 0.969029, as shown in Table 6. These findings highlight the LWSSA-MLP model’s ability to achieve a high degree of accuracy and reliability. Furthermore, the LWSSA-MLP model demonstrates significantly improved RMSE values compared to alternative frameworks, which is particularly noteworthy. During the learning phase, the MSE for LWSSA-MLP is calculated at 0.048447, while it achieves an MSE of 0.045385 during the testing phase. These results indicate that the LWSSA-MLP effectively minimizes prediction errors, outperforming other models analyzed in this study. Conclusively, the LWSSA-MLP model showcases a marked improvement in both error metrics and predictive precision. Its ability to enhance the MLP’s accuracy in estimating CO_2_ emission levels underscores its potential as a robust framework for addressing environmental degradation challenges.

**Table 5:** Training Results of Optimizer-based MLP Models.

Model	R^2^	RMSE	MSLE	MAE	MAPE
EDO-MLP	0.889406	0.094487	0.004307	0.070977	0.841958
LWSSA-MLP	**0.974899**	**0.048447**	**0.000916**	0.038289	0.435649
MFO-MLP	0.954088	0.065521	0.001868	0.049655	0.184302
SSA-MLP	0.960668	0.060645	0.001829	0.050948	1.452752
TSO-MLP	0.972769	0.050461	0.001123	0.038808	**0.158316**
YDSE-MLP	0.923109	0.078786	0.003002	0.063656	0.872722
MLP	0.905426	0.094039	0.003886	0.072181	2.367216
RWGWO-MLP	0.951567	0.062529	0.002123	0.047031	0.234546
AVOA-MLP	0.967993	0.050831	0.000976	**0.036251**	0.165402
GRO-MLP	0.934530	0.072699	0.002052	0.054381	0.210376
HBA-MLP	0.897429	0.090996	0.003582	0.067897	0.177824

Bold represent the best values in the table.

**Table 6:** Testing Results of Optimizer-based MLP Models.

Model	R^2^	RMSE	MSLE	MAE	MAPE
EDO-MLP	0.913295	0.095613	0.00505	0.080611	5.405761
LWSSA-MLP	**0.969029**	**0.045385**	**0.000746**	**0.03573**	**0.060454**
MFO-MLP	0.917055	0.074273	0.002488	0.060413	0.132387
SSA-MLP	0.924609	0.07081	0.002582	0.054552	0.108354
TSO-MLP	0.946231	0.0598	0.001717	0.048451	0.096465
YDSE-MLP	0.917537	0.093245	0.004769	0.079968	5.023884
MLP	0.892617	0.084509	0.002259	0.065382	0.100891
RWGWO-MLP	0.950289	0.072397	0.003123	0.049353	0.187967
AVOA-MLP	0.966094	0.059791	0.001591	0.045396	0.170198
GRO-MLP	0.936792	0.081636	0.002965	0.060765	0.231769
HBA-MLP	0.925878	0.088403	0.003558	0.066283	0.263338

Bold represent the best values in the table.

**Fig. 12:**
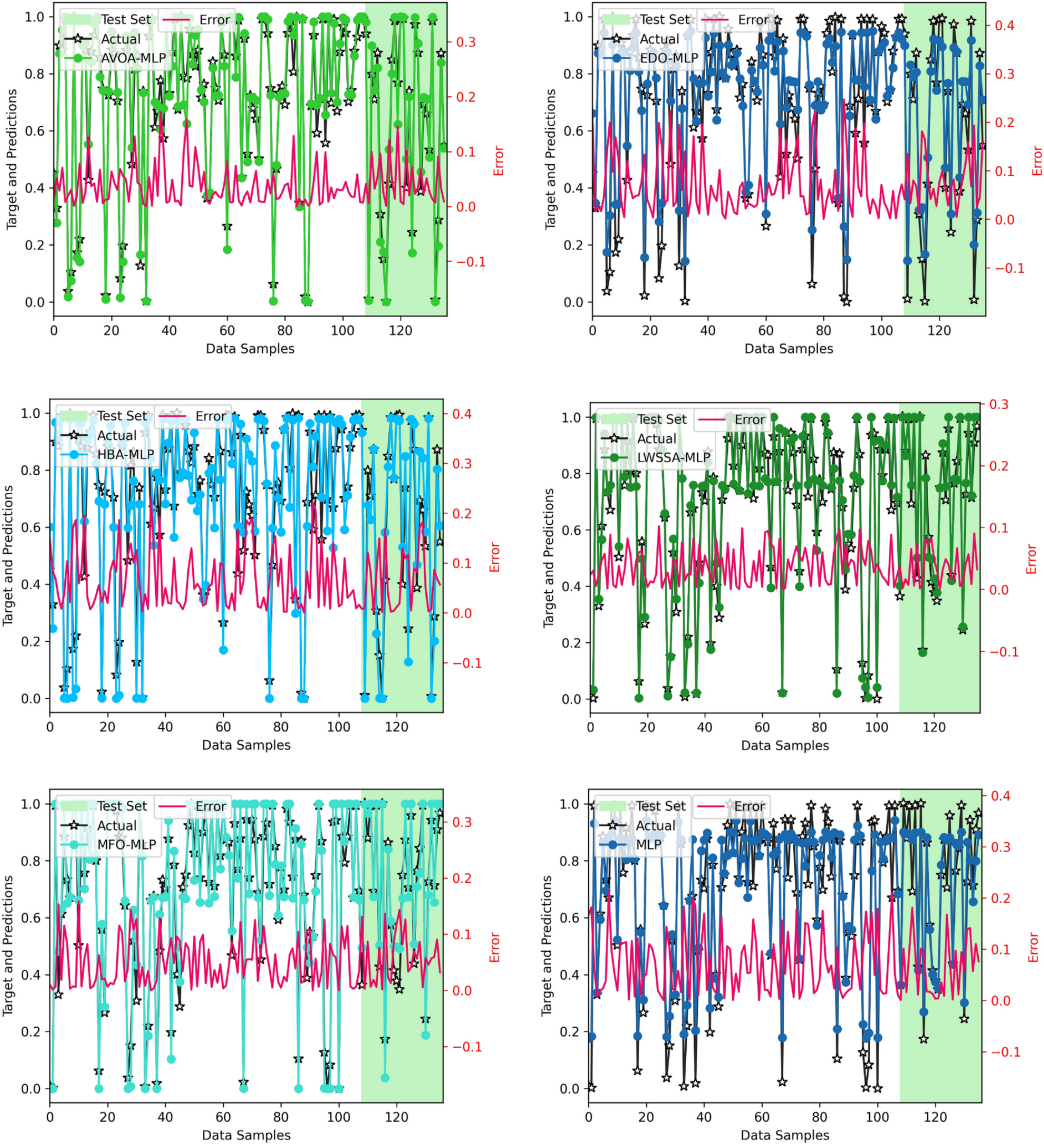

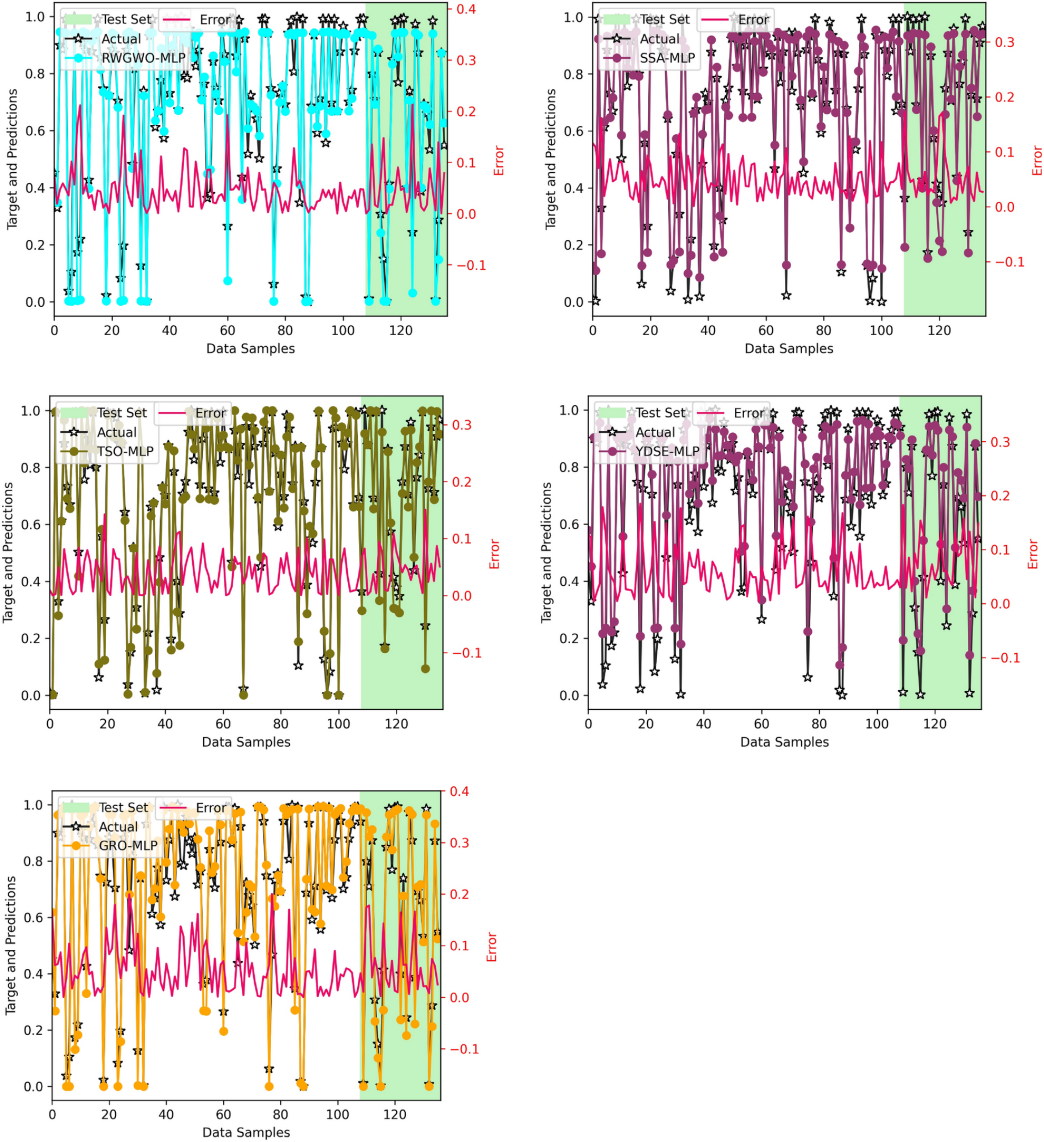
Absolute Error Plots of Optimizer-based MLP Models.

Figure 12 presents a comparative line plot analysis of actual CO₂ observed and the predicted CO₂ outcomes by various MLP algorithm-enhanced frameworks during both the training (learning) and testing (approximation using unseen data) stages. The pink lines in the figure represent the absolute error rate for all techniques. Notably, the predicted CO₂ levels align closely with the observed values for most frameworks, demonstrating their overall effectiveness. However, the LWSSA-MLP model stands out due to its exceptional consistency across both stages, as evidenced by its nearly horizontal error line, indicating minimal deviation. The LWSSA-MLP framework achieves superior accuracy in CO₂ predictions, attributed to its combinational optimization strategy (LWM and MM) that effectively balances exploitation and exploration. This equilibrium enables the framework to navigate the complexities of CO₂ prediction efficiently, resulting in precise and reliable prediction. In contrast, other frameworks exhibit varying levels of error. Notably, the EDO-MLP, MFO-MLP, HBA-MLP, and YDSE-MLP models display comparatively higher error rates, suggesting deficiencies in their ability to balance exploration and exploitation. This imbalance leads to suboptimal model performance, manifesting as underfitting or overfitting of data points. Among these, the EDO-MLP and YDSE-MLP frameworks exhibit the largest error rates, indicating significant challenges in accurately predicting CO₂ levels. This can be attributed to their inefficiency in managing the trade-off between exploration and exploitation, resulting in ineffective searches and erroneous predictions. These findings highlight the robust performance of LWSSA-MLP in addressing the intricate challenges of CO₂ prediction, setting it apart as a reliable and precise modeling framework.

**Fig. 13:**
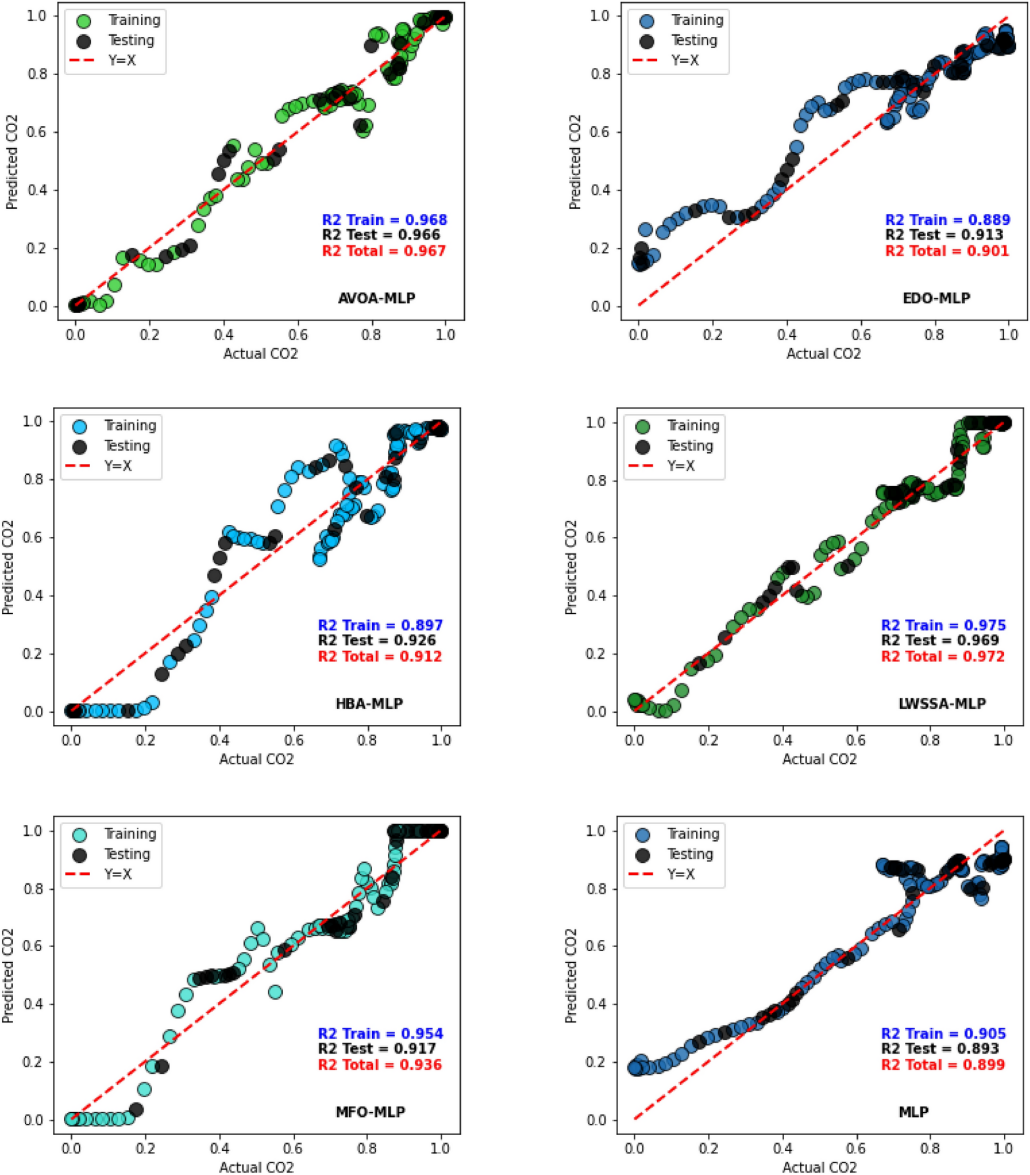

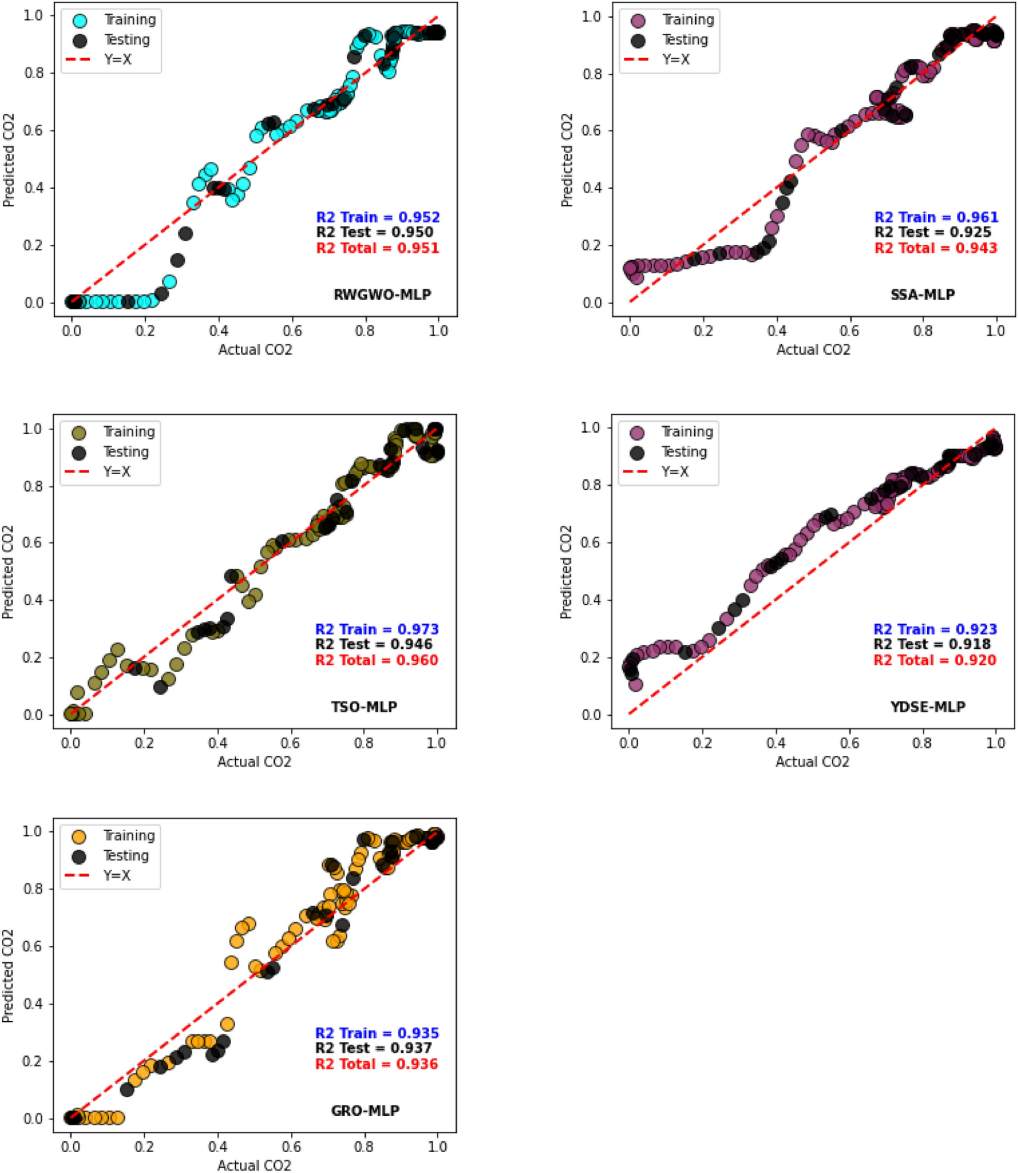
Actual vs Predicted Plots of Optimizer-based MLP Models.

Figure 13 provides scatter plot graphs of the actual CO₂ values and predicted values by various MLP optimizer-enhanced models. The R^2^ values for each technique are presented for both training and testing datasets, accompanied by plots comparing the predicted values with the actual observations. The red dotted line in the plot represents the ideal relationship, where predicted values perfectly align with their corresponding actual values.

The plot reveals that most MLP models show acceptable alignment with the ideal line, indicating superior performance. However, the LWSSA-MLP model distinctly outperforms all other frameworks, with its predictions consistently aligning closely with the ideal line. This outstanding performance is underscored by the highest R^2^ value among all models, an impressive 0.972, corresponding to a remarkable precision of 97.2% in matching real and predicted CO₂ values. The LWSSA-MLP model effectively captures underlying trends in the data, resulting in highly precise CO₂ prediction. In comparison, the TSO-MLP and SSA-MLP models exhibit lower R^2^ values than LWSSA-MLP, exhibiting a lack of effectiveness in uncovering hidden patterns within the dataset. Other models demonstrate varying levels of R^2^, with MFO-MLP, EDO-MLP, and YDSE-MLP showing the lowest values. These results indicate that these frameworks struggle to identify subtle trends in the data, leading to less accurate predictions. The findings show that the LWSSA-MLP model establishes itself as the definitive benchmark for CO₂ prediction in this study. Its unparalleled ability to reveal latent trends in the data and deliver highly accurate predictions highlights its superior performance. The model’s maximum R^2^ value and consistent alignment with the ideal line underscore its exceptional capability and precision, cementing its status as the most effective solution for R^2^ prediction .

The integration of optimization algorithms with machine learning models has demonstrated exceptional success in enhancing the predictive accuracy of CO₂ emission models. Recent advancements provide a robust foundation for comparing the performance of our proposed MLP-LWSSA model against existing methodologies. Moayedi et al. proposed an ANN optimized using Teaching–Learning-Based Optimization (TLBO) and Vortex Search (VS) algorithms, achieving MSE of 3.6778 during training^19^. Similarly, Luo et al. introduced a composite model that synergized various algorithms to predict carbon emissions with MAPE below 3.5%, a benchmark indicative of high reliability^49^. In addition, Yue et al. employed the Generalized Regression Neural Network (GRNN) enhanced by the Fruit Fly Optimization Algorithm (FOA), with the FOA-GRNN variant achieving a testing RMSE of 1.2492, marking significant improvements over traditional approaches^50^. Furthermore, Foong et al. leveraged Moth-Flame Optimization (MFO) in conjunction with Random Forest (RF), achieving RMSE values of 11.7065 and 12.8890 for testing and training, respectively. This innovative combination demonstrated substantial enhancements in predictive performance compared to conventional models^51^. These models underscore the efficacy of hybrid techniques in achieving superior predictive capabilities relative to traditional standalone methods. The proposed MLP-LWSSA model in this research showcases a highly competitive performance, with an R^2^ of 0.974899, RMSE of 0.048447, and MAPE of 0.435649 during training, and an R^2^ of 0.969029, RMSE of 0.045385, and MAPE of 0.060454 during testing. Our approach achieves an optimal balance between accuracy, minimal errors, and robustness, emphasizing its reliability and practical applicability compared to existing models. This positions the MLP-LWSSA model as a compelling contribution to the field, reinforcing the potential of optimization-integrated machine learning frameworks in addressing the critical challenge of CO₂ emission prediction.

#### Interpretation of feature importance scores of LWSSA-MLP

**Fig. 14:**
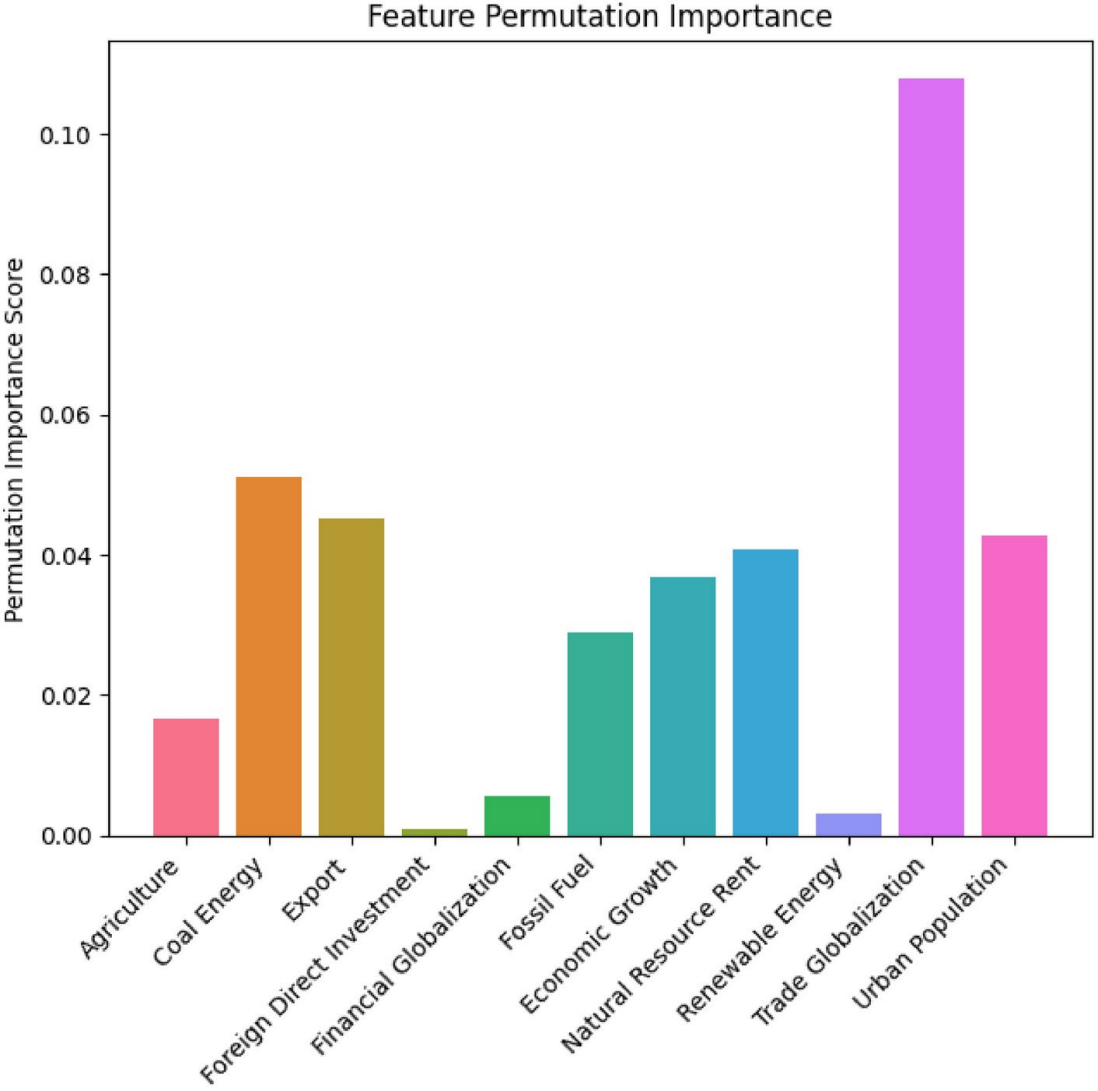
Features Significance Score.

Feature permutation importance measures the effect of each feature on the predictive power of the model. A higher importance score indicates a more significant contribution to CO_2_ emissions prediction. The feature permutation score is achieved by evaluating how much the performance of the model decreases when the values of a single feature are randomly shuffled. In this case, the metric observed is MSE. In other words, how the prediction error increases as the values of the feature are shuffled. This method provides a straightforward way to understand the influence of each feature on the model’s predictions. In the section, a detailed breakdown of the permutation importance scores for each feature used in our LWSSA-MLP model is presented in Fig. 14.

From Fig. 14, Trade Globalization holds the highest importance (0.1078), suggesting that the interconnectedness of global trade significantly influences CO_2_ emissions. The movement of goods and services on an international scale drives industrial activities and transportation, major sources of CO_2_ emissions. This agrees with studies that indicate that increased trade openness often leads to higher emissions due to the scale effect, where increased economic activity leads to higher energy consumption and emissions^52,53^. Coal energy’s significant impact reflects its status as one of the dirtiest energy sources. The combustion of coal for electricity and heat is a major source of CO_2_, and its high importance score (0.0510) in this study underscores the urgent need to reduce coal dependency. The detrimental effects of coal on the environment have been well-documented, with coal-fired power plants contributing approximately 30% of global CO_2_ emissions, as reported by the International Energy Agency in 2019. Both features, Export and Urban Population, are critical, highlighting the role of industrial output and urbanization in driving emissions with 0.0452 and 0.0427 feature importance scores, respectively. Industrial activities linked to exports can lead to increased energy consumption and CO_2_ emissions. Similarly, urbanization leads to higher energy demands and emissions from transportation, construction, and residential energy use^54,55^.

Natural Resource Rent importance score (0.0409) reflects the economic dependence on natural resources, correlating with possible extraction and processing activities that contribute to CO_2_ emissions. Resource extraction often involves significant energy use and environmental degradation. Economic Growth activities typically drive energy consumption as reflected by the feature importance score of 0.0368, which in turn impacts CO_2_ emissions. The relationship between economic growth and emissions is complex, involving both scale and technique effects, where the former increases emissions and the latter can potentially decrease through efficiency improvements^56,57^. The importance score of (0.0291) fossil fuel consumption underscores its direct link to CO_2_ emissions. Fossil fuels, including oil and natural gas, remain primary energy sources globally, and their combustion is a leading cause of anthropogenic CO_2_ emissions^58,59^. Agricultural practice’s impact on CO_2_ prediction in the dataset can be seen in the feature score of 0.0167. Agriculture contributes to emissions through mechanisms like deforestation, methane production from livestock, and nitrous oxide emissions from fertilized soils. While less significant than energy-related factors, the agricultural sector still plays a notable role in the carbon cycle and emissions.

Financial Globalization and Renewable Energy show less impact on the model’s prediction power, giving their low feature score 0.0057 and Renewable Energy 0.0031, respectively. These features have lower importance scores but still play a vital role. Financial globalization influences industrial growth and environmental policies, while renewable energy adoption helps offset emissions from fossil fuels. The transition to renewable energy is crucial for achieving emission reduction targets, although its current impact is less prominent compared to fossil fuels. The Foreign Direct Investment has a feature importance score of 0.0008. This feature has the least impact, suggesting that direct investments from abroad have a minor influence on CO_2_ emissions in this model. Finally, The importance scores indicate that global economic activities, energy consumption patterns such as coal, exportation, urbanization, and natural resources are primary drivers of CO_2_ emissions and have a huge impact on the models’ predictive power. Understanding the impact of these features on CO_2_ predictions allows for targeted interventions. For instance, reducing reliance on coal energy and enhancing the adoption of renewable energy sources could lead to substantial emission reductions. Additionally, managing the effects of urbanization and global trade can contribute to sustainable development goals by mitigating the environmental footprint of economic activities. The environmental implications of these findings are profound. Addressing the high-impact areas identified by the feature importance scores can lead to significant reductions in CO_2_ emissions, thereby contributing to global sustainability efforts. This aligns with the goals set by international agreements such as the Paris Agreement, which aims to limit global warming to well below 2 degrees Celsius.

#### Recommendations

Based on the findings, the following recommendations are proposed to mitigate CO_2_ emissions effectively:

1. Promote Renewable Energy: Given the lower importance score of renewable energy, increasing its share in the energy mix can offset the high impact of coal energy and fossil fuels on emissions. Policies that support the development and deployment of renewable energy technologies are essential. Studies have shown that renewable energy can significantly reduce greenhouse gas emissions when integrated into the energy system^60,61^.
2. Regulate and Optimize Trade Globalization: Implementing policies that promote sustainable practices in international trade can reduce the carbon footprint associated with the movement of goods. Encouraging green logistics, enhancing energy efficiency in transportation, and promoting the use of low-carbon technologies in trade activities are vital measures^62,63^.
3. Enhance Urban Planning: Developing sustainable urban infrastructure can mitigate the impact of urban population growth on emissions. This includes promoting public transportation, green buildings, and smart city initiatives that reduce energy consumption and emissions^64,65^.
4. Support Economic Diversification: Reducing economic dependence on natural resource rent and promoting diversified, low-carbon economic activities will lower emissions. Encouraging industries that are less carbon-intensive and fostering innovation in green technologies can drive sustainable economic growth.

In conclusion, addressing the high-impact areas identified by the feature importance scores will significantly reduce CO_2_ emissions, thereby contributing to global sustainability efforts. These targeted strategies will help align economic growth with environmental conservation, ensuring a balanced approach to development and ecological preservation.

## Conclusion

This study introduced an innovative LWSSA-MLP framework to enhance the accuracy and reliability of CO₂ emission predictions. By integrating the MLP with the LWSSA, the framework tackled the limitations of MLP, such as unfine-tuned parameters and reduced precision. The LWSSA enhanced the exploration and exploitation capabilities of the optimization process through LWM and MM, ensuring robust and efficient learning. The proposed LWSSA was compared with various optimizers to establish its superiority on the CEC2015 benchmark. The LWSSA-MLP framework demonstrated superior predictive performance, achieving an R^2^ value of 0.9749 and outperforming existing methods in key metrics such as RMSE, MSLE, MAE, and MAPE. A permutation feature significance analysis identified global trade, coal energy, export levels, urbanization, and natural resources as the most influential factors affecting CO₂ emissions. These insights provide a foundation for policymakers to prioritize mitigation strategies and develop targeted interventions.

While the results are promising, the study acknowledges limitations related to dataset diversity and regional adaptability. Future research will focus on expanding the framework’s applicability by incorporating diverse datasets and adapting it to region-specific characteristics. Additionally, exploring the dynamic interplay between CO₂ emissions and evolving socioeconomic variables will further enhance the framework’s predictive capabilities. This research underscores the potential of hybrid ML-optimization models in addressing complex environmental challenges. The proposed LWSSA-MLP framework serves as a powerful framework for predicting CO₂ emissions, enabling evidence-based decision-making to combat climate change and promote sustainable development. Future advancements aim to refine the framework’s accuracy and scalability, ensuring its relevance in a wide range of applications and global contexts.

## Data Availability

The data will be made available at reasonable request from the corresponding author.
